# Supplementary material for: Single-cell transcriptomics of human iPSC differentiation dynamics reveal a core molecular network of Parkinson’s disease
Source: Commun Biol. 2022 Jan 13;5:49. doi: 10.1038/s42003-021-02973-7 (PMC8758783; doi:10.1038/s42003-021-02973-7)
Supplement: Supplementary file 2 — Supplementary Information [file 42003_2021_2973_MOESM2_ESM.pdf]

Supplementary material to:

Single-cell transcriptomics of human iPSC differentiation dynamics reveal a core molecular network of Parkinson's disease

Gabriela Novak[1+,2,3]*, Dimitrios Kyriakis[1], Kamil Grzyb[1], Michela Bernini[1], Sophie Rodius[4], Gunnar Dittmar[4,5], Steven Finkbeiner[3], and Alexander Skupin[1,6]*

1. The Integrative Cell Signalling Group, Luxembourg Centre for Systems Biomedicine (LCSB), University of Luxembourg, Esch-sur-Alzette, Luxembourg.
2. Luxembourg Institute of Health (LIH), Esch-sur-Alzette, Luxembourg
3. Gladstone Institutes and Neurology and Physiology Department, UC San Francisco San Francisco, CA 94158, USA
4. Quantitative Biology Unit, Luxembourg Institute of Health, Strassen, Luxembourg
5. Department of Life Sciences and Medicine, University of Luxembourg, L-4367 Belvaux, Luxembourg
6. University of California San Diego, La Jolla, CA 92093, USA

\+ Primary affiliation

* Corresponding authors, email: gabriela.novak@alumni.utoronto.ca; alexander.skupin@uni.lu

Day -1	Plate iPSCs at 1.5 to double confluent density in MTeSR with ROCK inhibitor, remove ROCK inhibitor afer ±8 hours
Day 0	SRM, LDN193189 (100nM), SB431542 (10 µM)
Day 1-2	SRM, LDN193189 (100nM), SB431542 (10 µM), SHH (100ng/ml, Purmorphamine (2 µM), FgF-8b (100ng/ml)
Day 3-4	SRM, LDN193189 (100nM), SB431542 (10µM), SHH (100ng/ml), Purmorphamine (2µM), FgF-8b (100ng/ml), CHIR (3µM)
Day 5-6	75% SRM/25% N2 with LDN193189 (100nM), SHH (100ng/ml), Purmorphamine (2 µM), FgF-8b (100ng/ml), CHIR (3 µM)
Day 7-8	50% SRM/50% N2 with LDN193189 (100nM), SHH (100ng/ml), CHIR (3 µM)
Day 9-10	25% SRM/75% N2 with LDN193189 (100nM), SHH (100ng/ml), CHIR (3 µM)
Day 11-12	NB/B27, CHIR (3 µM), BDNF (20ng/ml), AA (0.2mM), GDNF (20ng/ml), cAMP (1mM), TGFB3 (1ng/ml), DAPT (10 µM)
Day 13-20	NB/B27 with with BDNF (20ng/ml), AA (0.2mM), GDNF (20ng/ml), cAMP (1mM), TGFB3 (1ng/ml), DAPT (10 µM)
Day 21	Dissociate using Accutase and passage 1:1 onto poly-L-ornithine/fibronectin/laminin-coated dishes.
Day 25	Passage again using Accutase, onto to poly-L-ornithine/fibronectin/laminin-coated dishes at about 3x10e6 cells/6-well well
Day 26+	NB/B27 with BDNF (20ng/ml), AA (0.2mM), GDNF (20ng/ml), cAMP (1mM), TGFB3 (1ng/ml), DAPT (10 µM)
SRM media contains 410 mL of Knockout DMEM (Invitrogen; cat. no. 10829-018), 75 mL Knockout Serum Replacement (Invitrogen, cat. no. 10828-028), 5 mL L-glutamine (200 mM, Invitrogen, cat. no. 25030-081), 5 mL MEM NEAA (Invitrogen, cat. no. 11140-050), and 0.5 mL of 1000x 2-mercaptoethanol (Invitrogen, cat. no. 21985-023).	
N2 media contains 470 mL of Neurobasal media, 5 mL L-glutamine (200 mM, Invitrogen, cat. no. 25030-081), N2 supplement (17502048), B27 supplement (12587010), 1% penicillin/streptomycin	
NB/B27 media contains 470 mL of Neurobasal media (Life Technologies Europe, 21103049), B27 supplement, 1% penicillin/streptomycin	

Supplementary Table 1. The mDA differentiation protocol. Timing of the differentiation protocol and differentiation factor concentrations used. SRM, N2 and NB/B27 refers to media protocols listed at the bottom of the table. Please note that significant modifications were made to the original protocol published by Kriks, S. et al.[1–3]

Differentiation timing and sample collection

Control cell line					PINK1 cell line				
				Plating					Plating
				Recovery					Recovery
				**D0**					**D0**
				D1					D1
				D2					D2
				D3					D3
			Plating	D4				Plating	D4
			Recovery	D5				Recovery	D5
			**D0**	D6				**D0**	D6
			D1	D7				D1	D7
			D2	D8				D2	D8
		Plating	D3	D9			Plating	D3	D9
		Recovery	D4	D10			Recovery	D4	D10
		**D0**	D5	D11			**D0**	D5	D11
		D1	D6	D12			D1	D6	D12
	Plating	D2	D7	D13		Plating	D2	D7	D13
	Recovery	D3	D8	D14		Recovery	D3	D8	D14
	**D0**	D4	D9	D15		**D0**	D4	D9	D15
	D1	D5	D10	D16		D1	D5	D10	D16
	D2	D6	D11	D17		D2	D6	D11	D17
	D3	D7	D12	D18		D3	D7	D12	D18
Propagated	D4	D8	D13	D19	Propagated	D4	D8	D13	D19
iPSC culture	D5	D9	D14	D20	iPSC culture	D5	D9	D14	D20
**iPSC**	**D6**	**D10**	**D15**	**D21**	**iPSC**	**D6**	**~~D10~~**	**D15**	**D21**

Collection of samples & single cell analysis

Supplementary Table 2. Timeline of sample generation and collection. See Supplementary Fig. 1 for differentiation protocol from D0 to D21. Between plating for differentiation, iPS cells were maintained in the MTeSR media (STEMCELL Technologies Inc.) and expanded to generate a sufficient number of cells for the next plating round. At each collection point, three 12-well plate wells were collected for qPCR (as independent biological replicates) and fourth was used for single-cell analysis (sc-RNAseq). Additional two wells were replated at D21, one at (1:1) and one onto a 96-well plate, the 96-well plate was fixed and stained at D25. At D25 the last well was replated onto a second 96-well plate, then fixed and stained at D35. Each sample was differentiated independently, as an independent biological replicate, from cells of a different passage number, since these were propagated in culture, until the next starting point.

mDA differentiation stages			
Stage 1	Stage 2	Stage 3	Stage 4
**Regional specification**	**Fllor Plate VM DA NPs**	**Differentiating VM DA neurons**	**Differentiated mDAs**
OTX2	LMX1A	NGN2 (Neurog)	SLC18A2 (VMAT2)
WNT1	FOXA2	NR4A2 (Nurr1)	SLC6A3 (DAT)
EN 1/2	MSX1	PITX3	ALDH1A1
LMX1B		TH	KCNJ6 (GIRK2)
			SNCA (a-synuclein)

Expression modules				
**IPSCs**	**Rgl**	**Prog**	**NProg**	**DA**
POU5F1	FABP7	SOX2	SOX2	FOXA2
L1TD1	SOX2	FOXA2	FOXA2	LMX1A
USP44	FOXA2	LMX1A	OTX2	TBB3
TDGF-1	LMX1A	OTX2	WNT5A	DDC
POLR3G	OTX2	WNT5A	ASCL1	DCX
TERF1	WNT5A	CORIN	NEUROD1	NR4A2
IFITM1	RSPO2	DDC	NEUROG2	PBX1
MYC	MSX1		TBB3	PITX3
	SOX6		DDC	EN1
	FABP7		DCX	TH
	CORIN			BNC2
	BNC2			SLC18A2
				SLC6A3
				LM03
				ALDH1A1
				SOX6

Supplementary Table 3. Key pathways expressed during the *in-vivo* mDA differentiation process. The mDA differentiation stages: key genes expressed at distinct stages of mDA neuron differentiation is based on work of Based on by Blaess & Ang[45], Bjorklund & S. B. Dunnet[6], Tiklova, Bjorklund et al.[7], Hegarty[8], and Arenas[9]. These mDA differentiation stage markers, which are specific and essential for mDA differentiation, were used to confirm that the correct mDA differentiation path was followed in vitro.
Expression modules: iPSCs (undifferentiated stem cells), radial glia (Rgl), progenitors leading to various cell types of the midbrain (Prog), to neural progenitors (NProg) which lead to mDA neurons, mDA neurons (DA). These groups are based on a recent publication by Ásgrímsdóttir & Arenas[10] and allowed us to show the progression of the differentiation process.

Gene	Max p-val.	Avg	Gene	Max p-val.	Avg	Gene	Max p-val.	Avg
SNHG5	2,84E-40	1,2233	***DNAJC15***	2,13E-05	-0,2908	***YPEL1***	2,24E-03	0,1418
PGK1	8,78E-31	0,5781	***TTC3***	2,27E-05	-0,2084	***DPH3***	2,27E-03	0,1660
CALM2P2	1,65E-22	-0,4551	***MYL9***	2,32E-05	0,1653	***ZNF593***	2,38E-03	0,1409
MTRNR2L1	3,71E-21	-0,7731	***IPO7***	3,20E-05	0,1495	***SNHG8***	2,44E-03	0,2942
MYL6	1,24E-19	0,3070	***PTGR1***	3,38E-05	-0,2377	***RNF26***	2,65E-03	0,1134
S100A6	2,09E-17	-0,4393	***C14ORF119***	3,53E-05	0,2194	***SLC7A8***	2,66E-03	-0,2006
ZNF280D	2,49E-17	-0,4377	***RP11.288E14.2***	4,65E-05	-0,1661	***THUMPD3***	2,66E-03	0,2241
TYW3	1,73E-14	0,4748	***NSA2***	4,67E-05	-0,2075	***NUP62***	2,75E-03	0,1122
TCEAL5	6,78E-14	-0,3745	***SRA1***	5,11E-05	0,2118	***RTCB***	2,78E-03	0,1859
MT.ND5	3,70E-13	-0,2243	***ZNF75A***	6,89E-05	-0,1561	***EIF3A***	2,83E-03	0,1690
RP11.641D5.1	4,00E-13	-0,4508	***GTF2I***	7,03E-05	-0,2326	***CD164***	2,83E-03	0,2346
HMGN1P38	3,95E-12	-0,3460	***FAM208A***	7,79E-05	-0,1284	***IPO9***	2,84E-03	0,1533
CRYZ	1,36E-11	0,2732	***RP11.676M6.1***	9,09E-05	-0,1732	***FAM126A***	2,86E-03	0,2571
MORF4L1P1	1,39E-11	-0,4552	***HLA.B***	1,25E-04	0,1239	***IQCB1***	2,93E-03	0,1169
TCF25	5,14E-11	-0,2072	***SMARCB1***	1,47E-04	-0,1477	***HNRNPCP1***	2,93E-03	-0,1692
CRKL	1,44E-10	0,2138	***SEPT2***	1,52E-04	-0,1305	***HSD17B4***	2,97E-03	-0,1338
CYFIP1	1,47E-10	0,3021	***HIC2***	1,57E-04	0,1798	***CCNB1IP1***	3,00E-03	0,1361
PTMAP5	2,02E-10	-0,4196	***EIF5P1***	1,70E-04	-0,2675	***SHROOM3***	3,03E-03	0,1114
RP11.692N5.2	3,69E-10	-0,5485	***SNAP29***	2,12E-04	0,2991	***RP13.258O15.1***	3,39E-03	-0,2489
HSPA8	4,07E-10	0,4696	***ACTN1***	2,14E-04	0,2552	***PSMD7***	3,85E-03	0,1997
C6ORF48	5,37E-10	0,3012	***RRP7A***	2,19E-04	0,1897	***TMEM38B***	3,98E-03	-0,1816
RP11.475C16.1	8,30E-10	-0,3438	***EIF1AY***	3,64E-04	0,2735	***SRSF9***	4,05E-03	-0,1431
NLRP2	1,91E-09	-0,4704	***RP11.488C13.1***	3,83E-04	-0,4249	***MYO10***	4,23E-03	0,1666
CCDC144NL.AS1	2,27E-09	0,5870	***AZI2***	4,11E-04	-0,1967	***AK6***	4,24E-03	0,1088
ZNF37A	3,66E-09	0,2595	***THAP9.AS1***	4,34E-04	-0,1848	***LANCL1***	4,28E-03	0,1186
MALAT1	8,50E-09	-0,2594	***CTSB***	5,60E-04	0,1873	***RRAGD***	4,30E-03	0,1525
HNRNPC	1,95E-08	-0,2880	***HNRNPCP2***	5,82E-04	-0,2255	***PFKP***	4,80E-03	0,2555
CD59	1,96E-08	0,2756	***CNTNAP2***	6,80E-04	0,2072	***NONO***	4,82E-03	-0,1609
MINOS1P3	2,08E-08	-0,3666	***SH3YL1***	7,01E-04	-0,1055	***STK33***	4,87E-03	-0,1139
NIPA2	2,62E-08	0,2650	***IWS1***	7,26E-04	0,1393	***PDAP1***	4,93E-03	-0,1437
THAP7	2,82E-08	0,1644	***WBSCR22***	7,45E-04	0,1442	***KMT2A***	5,37E-03	0,1419
TUBGCP5	3,34E-08	0,2310	***CSNK1G3***	7,96E-04	0,1590	***CHCHD2***	5,40E-03	-0,1740
ZNF880	5,11E-08	0,2523	***TRA2A***	8,89E-04	-0,1567	***EGLN3***	5,56E-03	0,2413
RP4.765C7.2	5,86E-08	-0,4253	***ATG101***	9,22E-04	0,1437	***RP4.796I17.5***	5,89E-03	-0,2372
CBX3P9	1,01E-07	-0,3360	***GOPC***	9,27E-04	-0,2850	***IFI27L1***	6,12E-03	0,1175
EFCAB2	1,23E-07	-0,2189	***RAB4A***	9,50E-04	-0,1061	***TCTN3***	6,14E-03	0,1193
ADGRG7	2,23E-07	-0,2592	***PSMB5***	1,02E-03	0,2740	***TCEAL7***	6,36E-03	0,1544
COMT	2,24E-07	0,2857	***PCBP1***	1,10E-03	0,2692	***SLC25A4***	6,39E-03	0,3074
AC009245.3	2,69E-07	-0,3344	***RP11.64B16.2***	1,15E-03	-0,1741	***CMTM8***	6,62E-03	-0,1925
PGD	6,71E-07	0,2393	***BTN2A2***	1,35E-03	0,1140	***FZD7***	7,11E-03	0,1098
PALLD	1,89E-06	0,2538	***RFWD3***	1,37E-03	0,1330	***ZNF83***	7,35E-03	-0,1129
GPC3	2,07E-06	0,3866	***NDUFB11***	1,46E-03	-0,2012	***LINC00909***	7,53E-03	-0,1021
SMARCA4	2,97E-06	0,2588	***EIF3B***	1,52E-03	0,1427	***C21ORF91***	7,57E-03	0,1393
NME4	4,13E-06	-0,2830	***CBX1***	1,56E-03	-0,2092	***CEP350***	7,93E-03	0,1442
PKP2	4,26E-06	0,1924	***AK4***	1,80E-03	0,1560	***SLK***	8,24E-03	0,1787
MLF1	4,69E-06	-0,3043	***MRPL15***	1,83E-03	0,1734	***CSTB***	8,38E-03	0,2675
C19ORF53	5,42E-06	0,3207	***EEF1A1***	1,88E-03	-0,2174	***SC5D***	8,78E-03	0,1420
MED15	1,26E-05	0,2428	***PSMG3***	1,90E-03	-0,1677	***CAT***	9,46E-03	-0,1288
OSBPL8	1,56E-05	0,2914	***WDR34***	2,01E-03	0,1276	***NAA20***	9,72E-03	0,1481
SMARCE1P6	1,86E-05	-0,1960	***RBM12***	2,10E-03	0,1040	***MRPL17***	9,86E-03	0,1464
						WDR11	9,94E-03	0,1321

Supplementary Table 4. Group B, 151 genes. Using the maximum adjusted p-value in a pairwise combinations as adjusted p-value, and the average fold change that occurred in the pairwise comparison as fold change threshold, identified genes only dysregulated in the same direction at all timepoints. This analysis led to 151 DEGs, which include previously identified genes of Group A, and of which 65 were upregulated and 86 downregulated compared with controls (padj < 0.01 and FC > 0.1).

Gene	Max p-val.	Avg	Gene	Max p-val.	Avg	Gene	Max p-val.	Avg
MTRNR2L1	3,71E-21	-7,73E-01	*SMARCB1*	1,47E-04	-1,48E-01	*TCEAL7*	6,36E-03	1,54E-01
RP11.692N5.2	3,69E-10	-5,49E-01	*PDAP1*	4,93E-03	-1,44E-01	*AK4*	1,80E-03	1,56E-01
NLRP2	1,91E-09	-4,70E-01	*SRSF9*	4,05E-03	-1,43E-01	*CSNK1G3*	7,96E-04	1,59E-01
MORF4L1P1	1,39E-11	-4,55E-01	*HSD17B4*	2,97E-03	-1,34E-01	*THAP7*	2,82E-08	1,64E-01
CALM2P2	1,65E-22	-4,55E-01	*SEPT2*	1,52E-04	-1,30E-01	*MYL9*	2,32E-05	1,65E-01
RP11.641D5.1	4,00E-13	-4,51E-01	*CAT*	9,46E-03	-1,29E-01	*DPH3*	2,27E-03	1,66E-01
S100A6	2,09E-17	-4,39E-01	*FAM208A*	7,79E-05	-1,28E-01	*MYO10*	4,23E-03	1,67E-01
ZNF280D	2,49E-17	-4,38E-01	*STK33*	4,87E-03	-1,14E-01	*EIF3A*	2,83E-03	1,69E-01
RP4.765C7.2	5,86E-08	-4,25E-01	*ZNF83*	7,35E-03	-1,13E-01	*MRPL15*	1,83E-03	1,73E-01
RP11.488C13.1	3,83E-04	-4,25E-01	*GOLGA8B*	3,36E-03	-1,11E-01	*SLK*	8,24E-03	1,79E-01
PTMAP5	2,02E-10	-4,20E-01	*MGMT*	8,83E-03	-1,11E-01	*HIC2*	1,57E-04	1,80E-01
TCEAL5	6,78E-14	-3,75E-01	*RNPC3*	5,86E-03	-1,08E-01	*RTCB*	2,78E-03	1,86E-01
MINOS1P3	2,08E-08	-3,67E-01	*RAB4A*	9,50E-04	-1,06E-01	*CTSB*	5,60E-04	1,87E-01
PLCB4	6,73E-03	-3,61E-01	*SH3YL1*	7,01E-04	-1,06E-01	*RRP7A*	2,19E-04	1,90E-01
HMGN1P38	3,95E-12	-3,46E-01	*FBXO9*	1,03E-03	-1,03E-01	*PKP2*	4,26E-06	1,92E-01
RP11.475C16.1	8,30E-10	-3,44E-01	*LINC00909*	7,53E-03	-1,02E-01	*PSMD7*	3,85E-03	2,00E-01
CBX3P9	1,01E-07	-3,36E-01	*RBM12*	2,10E-03	1,04E-01	*CNTNAP2*	6,80E-04	2,07E-01
AC009245.3	2,69E-07	-3,34E-01	*SPRED1*	8,51E-05	1,05E-01	*SRA1*	5,11E-05	2,12E-01
MLF1	4,69E-06	-3,04E-01	*ABRACL*	3,57E-03	1,05E-01	*CRKL*	1,44E-10	2,14E-01
DNAJC15	2,13E-05	-2,91E-01	*CNN3*	2,49E-04	1,07E-01	*C14ORF119*	3,53E-05	2,19E-01
HNRNPC	1,95E-08	-2,88E-01	*AK6*	4,24E-03	1,09E-01	*THUMPD3*	2,66E-03	2,24E-01
GOPC	9,27E-04	-2,85E-01	*FZD7*	7,11E-03	1,10E-01	*TAGLN*	3,67E-03	2,31E-01
NME4	4,13E-06	-2,83E-01	*CUL3*	1,58E-06	1,10E-01	*TUBGCP5*	3,34E-08	2,31E-01
EIF5P1	1,70E-04	-2,68E-01	*SHROOM3*	3,03E-03	1,11E-01	*CD164*	2,83E-03	2,35E-01
RALGPS2	5,42E-04	-2,64E-01	*NUP62*	2,75E-03	1,12E-01	*PGD*	6,71E-07	2,39E-01
MALAT1	8,50E-09	-2,59E-01	*RNF26*	2,65E-03	1,13E-01	*EGLN3*	5,56E-03	2,41E-01
ADGRG7	2,23E-07	-2,59E-01	*BTN2A2*	1,35E-03	1,14E-01	*MED15*	1,26E-05	2,43E-01
RP13.258O15.1	3,39E-03	-2,49E-01	*IQCB1*	2,93E-03	1,17E-01	*ZNF880*	5,11E-08	2,52E-01
PTGR1	3,38E-05	-2,38E-01	*IFI27L1*	6,12E-03	1,17E-01	*PALLD*	1,89E-06	2,54E-01
RP4.796I17.5	5,89E-03	-2,37E-01	*LANCL1*	4,28E-03	1,19E-01	*ACTN1*	2,14E-04	2,55E-01
GTF2I	7,03E-05	-2,33E-01	*PTPRZ1*	2,10E-03	1,19E-01	*PFKP*	4,80E-03	2,56E-01
FOS	2,71E-03	-2,31E-01	*TCTN3*	6,14E-03	1,19E-01	*FAM126A*	2,86E-03	2,57E-01
HNRNPCP2	5,82E-04	-2,26E-01	*EXOC5*	3,74E-05	1,21E-01	*SMARCA4*	2,97E-06	2,59E-01
MT-ND5	3,70E-13	-2,24E-01	*HLA.B*	1,25E-04	1,24E-01	*ZNF37A*	3,66E-09	2,59E-01
EFCAB2	1,23E-07	-2,19E-01	*WDR34*	2,01E-03	1,28E-01	*NIPA2*	2,62E-08	2,65E-01
EEF1A1	1,88E-03	-2,17E-01	*CYCS*	5,25E-04	1,31E-01	*CSTB*	8,38E-03	2,68E-01
PHYH	6,13E-05	-2,17E-01	*WDR11*	9,94E-03	1,32E-01	*PCBP1*	1,10E-03	2,69E-01
CBX1	1,56E-03	-2,09E-01	*RFWD3*	1,37E-03	1,33E-01	*CRYZ*	1,36E-11	2,73E-01
TTC3	2,27E-05	-2,08E-01	*CCNB1IP1*	3,00E-03	1,36E-01	*EIF1AY*	3,64E-04	2,73E-01
NSA2	4,67E-05	-2,08E-01	*IWS1*	7,26E-04	1,39E-01	*PSMB5*	1,02E-03	2,74E-01
TCF25	5,14E-11	-2,07E-01	*C21ORF91*	7,57E-03	1,39E-01	*CD59*	1,96E-08	2,76E-01
NDUFB11	1,46E-03	-2,01E-01	*ZNF593*	2,38E-03	1,41E-01	*COMT*	2,24E-07	2,86E-01
SLC7A8	2,66E-03	-2,01E-01	*GNB1*	3,01E-03	1,41E-01	*OSBPL8*	1,56E-05	2,91E-01
RSRP1	2,91E-06	-1,98E-01	*YPEL1*	2,24E-03	1,42E-01	*SNHG8*	2,44E-03	2,94E-01
AZI2	4,11E-04	-1,97E-01	*KMT2A*	5,37E-03	1,42E-01	*SNAP29*	2,12E-04	2,99E-01
SMARCE1P6	1,86E-05	-1,96E-01	*SC5D*	8,78E-03	1,42E-01	*C6ORF48*	5,37E-10	3,01E-01
CMTM8	6,62E-03	-1,93E-01	*EIF3B*	1,52E-03	1,43E-01	*CYFIP1*	1,47E-10	3,02E-01
THAP9.AS1	4,34E-04	-1,85E-01	*FRA10AC1*	2,39E-04	1,43E-01	*MYL6*	1,24E-19	3,07E-01
TMEM38B	3,98E-03	-1,82E-01	*IPO5*	6,49E-03	1,43E-01	*SLC25A4*	6,39E-03	3,07E-01
RP11.64B16.2	1,15E-03	-1,74E-01	*ATG101*	9,22E-04	1,44E-01	*C19ORF53*	5,42E-06	3,21E-01
CHCHD2	5,40E-03	-1,74E-01	*CEP350*	7,93E-03	1,44E-01	*GPC3*	2,07E-06	3,87E-01
RP11.676M6.1	9,09E-05	-1,73E-01	*WBSCR22*	7,45E-04	1,44E-01	*HSPA8*	4,07E-10	4,70E-01
HNRNPCP1	2,93E-03	-1,69E-01	*MRPL17*	9,86E-03	1,46E-01	*TYW3*	1,73E-14	4,75E-01
PSMG3	1,90E-03	-1,68E-01	*NAA20*	9,72E-03	1,48E-01	*PGK1*	8,78E-31	5,78E-01
RP11.288E14.2	4,65E-05	-1,66E-01	*IPO7*	3,20E-05	1,50E-01	*CCDC144NL.AS1*	2,27E-09	5,87E-01
NONO	4,82E-03	-1,61E-01	*CENPW*	2,16E-03	1,50E-01	*SNHG5*	2,84E-40	1,22E+00
TRA2A	8,89E-04	-1,57E-01	*RRAGD*	4,30E-03	1,52E-01			
ZNF75A	6,89E-05	-1,56E-01	*IPO9*	2,84E-03	1,53E-01			

Supplementary Table 5. Group C, 172 genes. Repeating the same analysis for the four timepoints (iPSCs, D6, D15 and D21) as in Group B, but taking into account only the absolute degree of change in iPSCs, yielded 172 genes. The analysis is based on gene expression matrix of 4495 cells (39,194 genes). For Group D (292 genes) see Supplementary Data 1.

PARK1&4	*SNCA*
PARK2	*PARKIN*
PARK3	unknown / not found
PARK5	*UCHL1*
PARK6	*PINK1*
PARK7	*DJ-1*
PARK8	*LRRK2*
PARK9	*ATP13A2*
PARK10	*USP24*
PARK11	*GRB10*
PARK12	unknown / risk factor locus
PARK13	*HTRA2*
PARK14	*PLA2G6*
PARK15	*FBXO7*
PARK16	unknown / risk factor locus
PARK17	*VPS35*
PARK18	*EIF4G1*
PARK19	*DNAJC6*
PARK20	*SYNJ1*
PARK21	*DNAJC13*
PARK22	***CHCHD2***
PARK23	*VPS13C*

Supplementary Table 6. Parkinson's disease-associated (PARK) genes[11–17]. Variants in several genes or loci have been shown to either cause or be associated with increased susceptibility to PD, these have been designated the PARK loci/genes. CHCHD2 is highlighted in bold, as it is also a DEG gene identified by our SC-RNAseq analysis.

PCR primers		
**Gene**	**qRT-PCR primer forward**	**qRT-PCR primer reverse**
OTX2	GAGGTGGCACTGAAAATCAAC	TCTTCTTTTTGGCAGGTCTCA
EN1	CGTGGTCAAAACTGACTCGC	CGCTTGTCCTCCTTCTCGTT
LMX1A	GCTCAGAGCAGTTCAGAGGG	CAAGCAGGAGTTTGCCCAAC
FOXA2	ATTTTAAACTGCCATGCACTCG	ATGTTGCTCACGGAGGAGTAG
TH	GGAAATTGAGAAGCTGTCCACG	GAATCTCAGGCTCCTCAGACAG
ALDH1A1	GAGAGTGGGAAGAAAGAAGGGG	TTCATGATTTGCTGCACTGGTC
GIRK2/KCNJ6	AGCTGCCCAAAGAGGAACTG	ACAGGTGTGAACCGGTAACC
GAPDH	AAGAAGGTGGTGAAGCAGGC	GTCAAAGGTGGAGGAGTGGG
	**Sequencing primer forward**	**Sequencing primer reverse**
PINK1	GAGGTCCCAAGCAACTAGCC	TGGCCGTAGAAGGGATTGAC

**Antibodies**					
**Target**	**Host**	**reactivity**	**Manufacturer**	**Dilution**	**Catalogue #**
**1oAb**					
POU5F1 (Oct3/4)	mouse	human	Santa Cruz Biotechnology	1:500	sc-5279
Tra-1-60	mouse	human	Merck Millipore	1:500	MAB4360
PITX3	rabbit	human	sigmaaldrich	1:100	HPA044639
LMX1A	rabbit	human	abcam	1:500	ab139726
Slc6A3/DAT	rabbit	human	Thermofisher	1:1000	PA1-4656
MAP2	mouse	human & other	Sigma / Merck	1:500	MAB3418
TH	rabbit	human	Pel-Freez Biologicals	1:500	P40101
TH	mouse	human	Merck Millipore	1:500	MAB 318
**2oAb**					
Donkey anti-Rabbit IgG, Alexa Fluor 488			Thermofisher	1:1000	A32790
Donkey anti-Mouse IgG, Alexa Fluor Plus 555			Thermofisher	1:1000	A32773

Supplementary Table 7. List of primers used for qPCR expression analysis, sequencing and antibodies used for immunocytochemistry.

DAY 25	DAY 40
ABCA8	ALCAM
ATRIP	ANXA1
CACNA2D1	CD99
CRABP1	CPE
CSRP2	**DDC**
**DDC**	FAM127A
DPP7	MAOB
FABP7	**NES**
FARP1	PON2
FLNC	SEPT6
FUCA1	**TH**
KIF21A	**VIM**
MAP6	
MCAM	
MCM2	
MCM6	
MUT	
NEBL	
NENF	
**NES**	
PSAT1	
RTL1	
STXBP1	
**TH**	
TPBG	
TPPP3	
TSPAN6	
VAT1L	
**VIM**	
VWA5A	
WLS	

Supplementary Table 8. Proteomics analysis. Proteins differentially abundant in a PINK cell line, compared to a control, in two biological duplicates per each timepoint (D25 and D40). Proteins found differentially abundant at both timepoints are highlighted in bold. Proteins also identified as by SC-RNAseq as differentially expressed at the mRNA level are underlined.

Amp/Deletion Table									
Chr	Amp/Del	Start(bp)	Stop(bp)	Size(kb)	Chr Band	# Probes	Log2 Ratio	Genes *	Overlap Normal CNVs? +
2	AMP	188,058,313	189,724,370	1,666	q32.1 - q32.2	50	0.292488	CALCRL, TFPI, LINC01090, GULP1, No MIR561, DIRC1	No
3	AMP	146,708,800	147,362,016	653	q24	81	0.299431	ZIC4, ZIC1, LOC440982	No
4	AMP	61,619,455	63,761,135	2,142	q13.1	66	0.255822	MIR548AG1, ADGRL3, ADGRL3-AS1	No
4	AMP	92,202,997	94,796,142	2,593	q22.1 - q22.2	87	0.293805	CCSER1, LOC101929194, GRID2, ATOH1	No
5	AMP	146,716,607	147,447,900	731	q32	44	0.313221	STK32A, DPYSL3, JAKMIP2-AS1, JAKMIP2, SPINK1, SCGB3A2, C5orf46, SPINK5	No
7	AMP	100,980,697	101,126,393	146	q22.1	6	0.609012	COL26A1	Yes
7	DEL	110,564,801	110,708,070	143	q31.1	6	-0.9213	IMMP2L	Yes
7	AMP	121,256,234	122,628,757	1,373	q31.32	142	0.328262	PTPRZ1, AASS, FEZF1, FEZF1-AS1, CADPS2, RNF133, RNF148	No
9	DEL	43,590,480	43,841,603	251	p12 - p11.2	6	-0.74614	FAM74A7, SPATA31A6, CNTNAP3B, CNTNAP3P2	Yes
11	AMP	91,344,721	92,716,856	1,372	q14.3	45	0.302978	FAT3, LOC105369431, MTNR1B	No
12	DEL	92,022,671	93,355,847	1,333	q21.33 - q22	44	-0.25259	LINC01619, BTG1, LOC101928617, CLLU1OS, CLLU1, C12orf74, PLEKHG7, EEA1	No
14	AMP	20,203,610	20,421,677	218	q11.2	8	0.511181	OR4Q3, OR4M1, OR4N2, OR4K2, OR4K5, OR4K1	Yes
16	AMP	34,452,586	34,743,643	291	p11.2 - p11.1	12	0.371214	LINC01566, FRG2DP, TP53TG3HP	Yes
22	AMP	25,672,585	25,903,543	231	q11.23 - q12.1	9	0.568746	IGLL3P, LRP5L, CRYBB2P1, MIR6817	Yes
X	AMP	112,594,478	115,303,379	2,709	q23	100	0.280264	XACT, HTR2C, MIR448, IL13RA2, LUZP4, PLS3, AGTR2, LOC101928437, SNORA35, MIR764, MIR1912, MIR1264, MIR1298, MIR1911, LRCH2, RBMXL3, PLS3-AS1, DANT2	No
Total Amp/Del: 15									

Supplementary Table 9. Amplifications and Deletions. Data was obtained from a CLG Microarray test performed at passage 9 (P9) (Supplementary Fig. 2). * Genes amplified or deleted are cross referenced against the Online Mendelian Inheritance in Man (OMIM®) database.

LOH Intervals Table							
Chr	Start(bp)	Stop(bp)	Size(kb)	Chr Band	# Probes	LOH Score	Genes *
1	1,089,699	39,243,405	38,154	p36.33 - p34.3	402	36.74485	Too Numerous to List
3	123,562,478	127,688,648	4,126	q21.1 - q21.3	54	6.073903	Too Numerous to List
6	1,696,841	18,775,310	17,078	p25.3 - p22.3	385	28.04076	Too Numerous to List
7	79,723,943	89,361,740	9,638	q21.11 - q21.13	189	15.34061	GNAI1, GNAT3, CD36, SEMA3C, HGF, PCLO, SEMA3 E, SEMA3A, SEMA3D, GRM3, KIAA1324L, DMTF1, T P53TG1, CROT, ABCB4, ABCB1, SLC25A40, ADAM22 , SRI, STEAP4, LOC101927269, LOC100128317, CAC NA2D1,LOC101927356, LOC101927378, LINC00972 , TMEM243, RUNDC3B, DBF4, LOC102723885, ZNF 804B, C7orf62
16	55,121,927	84,673,276	29,551	q12.2 - q24.1	575	55.62815	Too Numerous to List
18	62,974,135	65,905,964	2,932	q22.1	77	6.992831	CDH7, CDH19, DSEL, MIR5011, LOC643542
20	39,476,952	42,671,183	3,194	q12 - q13.12	62	6.616215	TOP1, PLCG1, ZHX3, LPIN3, CHD6, PTPRT, SRSF6, L3MBTL1, SGK2, MYBL2, TOX2 , LOC100128988, PLCG1-AS1, MIR6871, EMILIN3, LOC101927159, IFT52, GTSF1L, LOC105372626
22	44,261,581	48,849,346	4,588	q13.31 - q13.32	76	6.992831	Too Numerous to List
Total LOH Intervals: 8							

Supplementary Table 10. LOH Intervals Table. Data was obtained from a CLG Microarray test performed at passage 9 (P9) (Supplementary Fig. 2). * Genes amplified or deleted are cross referenced against the Online Mendelian Inheritance in Man (OMIM®) database.

Cell Line GENETICS

Cell Line Characterization

Cell Line ID: ND 4066A
Passage #: 2
Specimen Type: Human Fibroblast Culture
Indication for Study: Routine Culture QC

Test Code: 100
Account #: NA
PO #: 135048

Date Received: 5/29/2015
Date Reported: 6/11/2015
Time in Culture: 1 Day

Lab #: CLG-20102
PI: Dr. Steven Finkbeiner
Contact Person: Gabriela Novak
Email: gabriela.novak@gladstone.ucsf.edu
Address:
Gladstone Institutes
1650 Owens Street
San Francisco, California 94158

Additional copies sent to:

Banding Technique: GTL **Band Resolution:** Fair
Metaphases Counted: 20 Analyzed: 7 Karyotyped: 2

RESULTS: 46,XY[20] Apparently NORMAL Human Male Karyotype

Non-clonal Aberrations: None

INTERPRETATION:

Cytogenetic analysis was performed on twenty G-banded metaphase cells from human cell line ND 4066A p2 and all twenty cells demonstrated an apparently normal male karyotype.

Supplementary Figure 1. Fibroblast ND40066 cytogenetic analysis.

Cell Line GENETICS

www.clgenetics.com
510 Charmany Drive • #254-255 • Madison, Wisconsin 53719 • 608-441-8160

CLG Microarray Test Results

Cell Line ID: ND 40066 clone8 p9 **Lab #:** CLG-24270 **Date received:** 6/17/16 **Date Reported:** 7/11/16

Contact Person: Gabriela Novak/Gaia Skibinski **PI:** Finkbeiner **Institute:** Gladstone

Test Code: aCGH 110 **Email:** gabriela.novak@gladstone.ucsf.edu/gaiaskibinski@gmail.com **PO #:** 135048

Mailing Address: 1650 Owens Street, San Francisco, CA 94158

Sample Type: Human iPSC Frozen Cells **dsDNA Concentration:** 454.0 ng/µl **Total dsDNA:** 5.9 ug

Sex: Male **260/280** (1.7-1.9): 1.8 **260/230** (≥1.90): 2.2 **Array Type:** Agilent 180K Standard aCGH

Array ID Number: 252983034296_1_2 **Reference DNA:** Agilent Euro Male

---

Quality Control

A sufficient amount of high quality genomic DNA, as determined by UV spec. (NanoVue), fluorometer (Qubit) and Agarose Gel analysis, was extracted from cell line ND 40066 clone 8 p9 and passed our internal quality standards for aCGH labeling.

aCGH Probes (PASS/FAIL): Pass
SNP Probes (PASS/FAIL): Pass

Experimental Deviations: None

Results:

Clonal Fraction: 100%

See attached sheets for Tabular and Graphical presentation of microarray results.

Variants are considered provisional until confirmed by another technique. For further confirmation of a particular variant, CLG recommends using Karyotyping (variants >5Mb), FISH (variants>200Kb).

Supplementary Figure 2. iPSC ND40066 clone 8 aCGH analysis performed at P9.

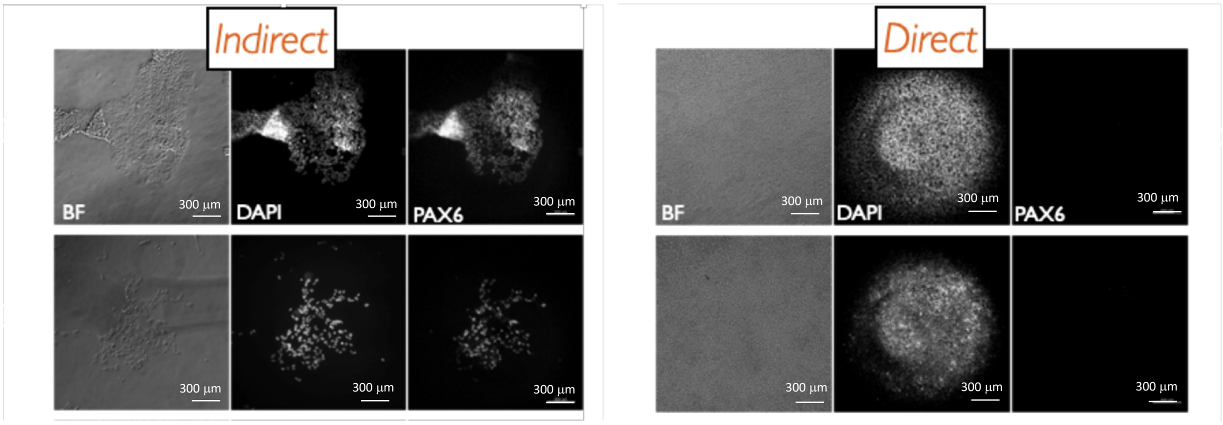

Supplementary Figure 3. Staining for the non-midbrain DA marker PAX6. “indirect” indicates indirect differentiation of DA neurons using rosettes in a two-stage process [9], while “direct” indicates mDA differentiation using our protocol which differentiates iPSC into mDA neurons using one continuous process [9,18].

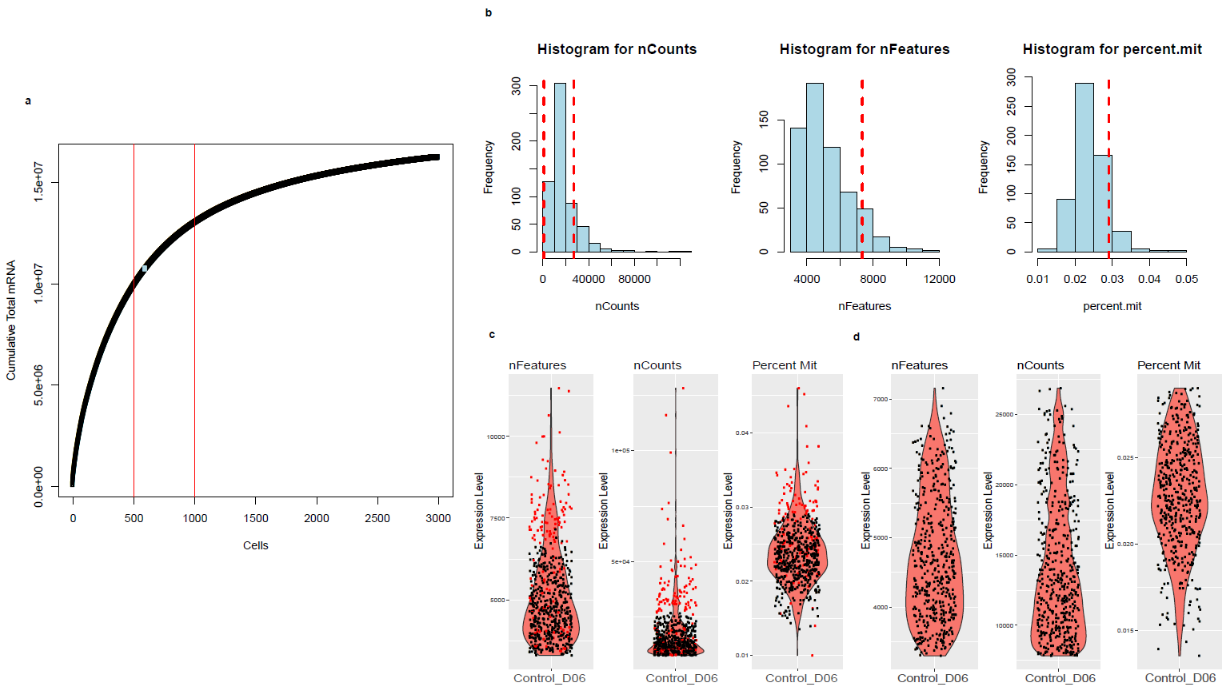

Supplementary Figure 4. Quality control Plots of control sample on Day 06. **a)**. Cumulative Total number of counts. The red vertical lines represent the down and upper bound of the expected elbow. The blue dot represents the transitional point calculated using ecp r package. **b).** Histograms of the three criteria that were used for low quality cell filtering. **c,d)** Violin plots showing the distribution of the three criteria (nFeature= number of features/genes. nCount= number of counts, Percent.Mit = mitochondrial counts percentage). Each dot represent a cell in the dataset. c). The distribution of the three metrics before filtering. Red dots are cells that filtered after the quality control and black dots are cells which were kept for downstream analysis. d) The distributions of the QC metrics after filtering step.

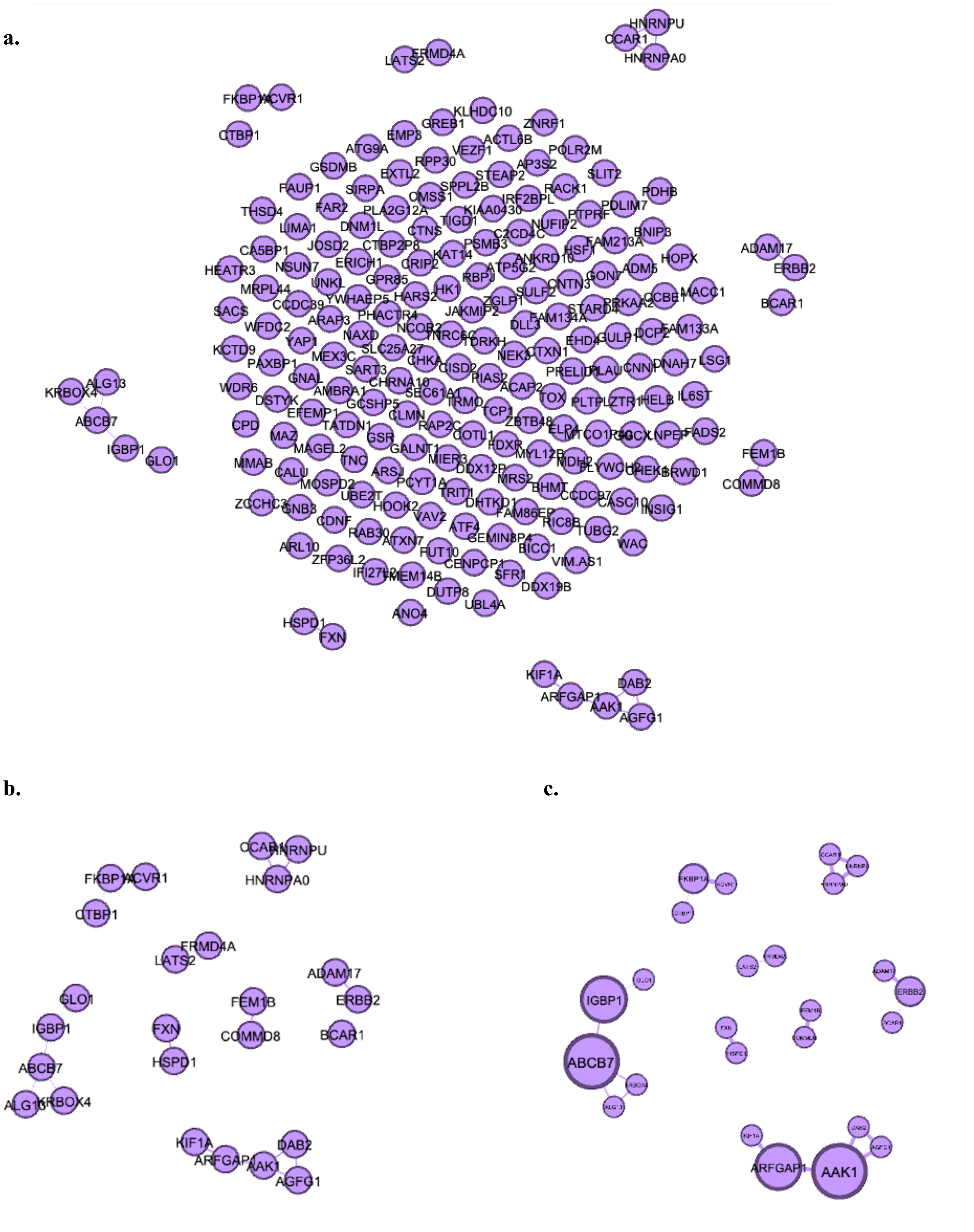

Supplementary Figure 5. Network analysis performed on randomly selected genes using Gephi. a). 200 randomly selected genes, processed the same way as DE genes. Central group consists of unconnected nodes. **b).** Unconnected nodes filtered out, only nodes with a conection to at least one other remain. **c).** Betweenness centrality applied, size reflects connectedness of nodes.

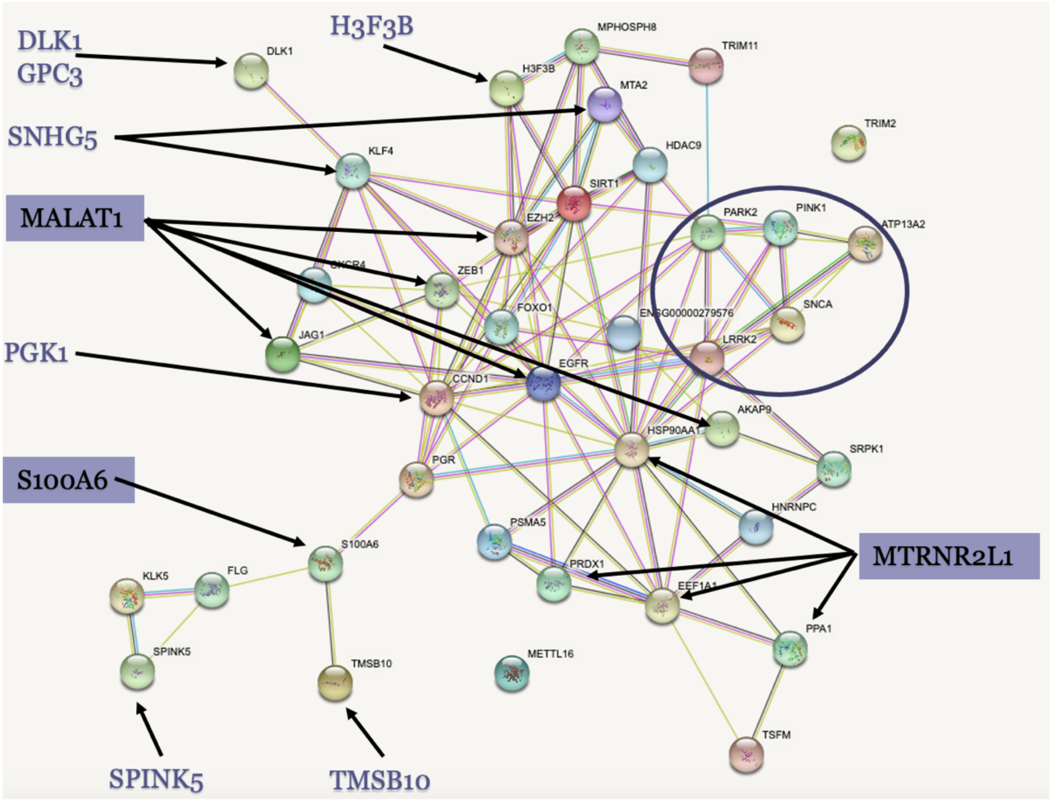

Supplementary Figure 6. The network of genes dysregulated by the presence of the PINK1 mutation includes genes related to other PD-associated pathways, based on the STRING[19] database network. GOPC and GPC3 [20,21] interact with the PD-associated gene DJ-1 (PARK7) [11,12]. The DEG network also includes genes of the LRRK2 (PARK8) network [11,12], namely ENAH, HSPA8, MYL6, **MALAT1**, and **SNHG5**. SNHG5 and MALAT1 interact with LRRK2 via miR-205-5p [44,45]. **DLK1** and **MALAT1** mediate α-synuclein accumulation [22,23]. In fact, the DLK1-NURR1 interaction involved in this process may be mDA neuron-specific [24], highlighting the necessity to use mDA neurons for the study of PD-related pathways. SNHG5 and MALAT1 are not included in the protein-protein interaction-based network analysis, since they are RNA genes and do not code for proteins. The involvement of non-coding RNA is likely yet another layer of PD pathology.

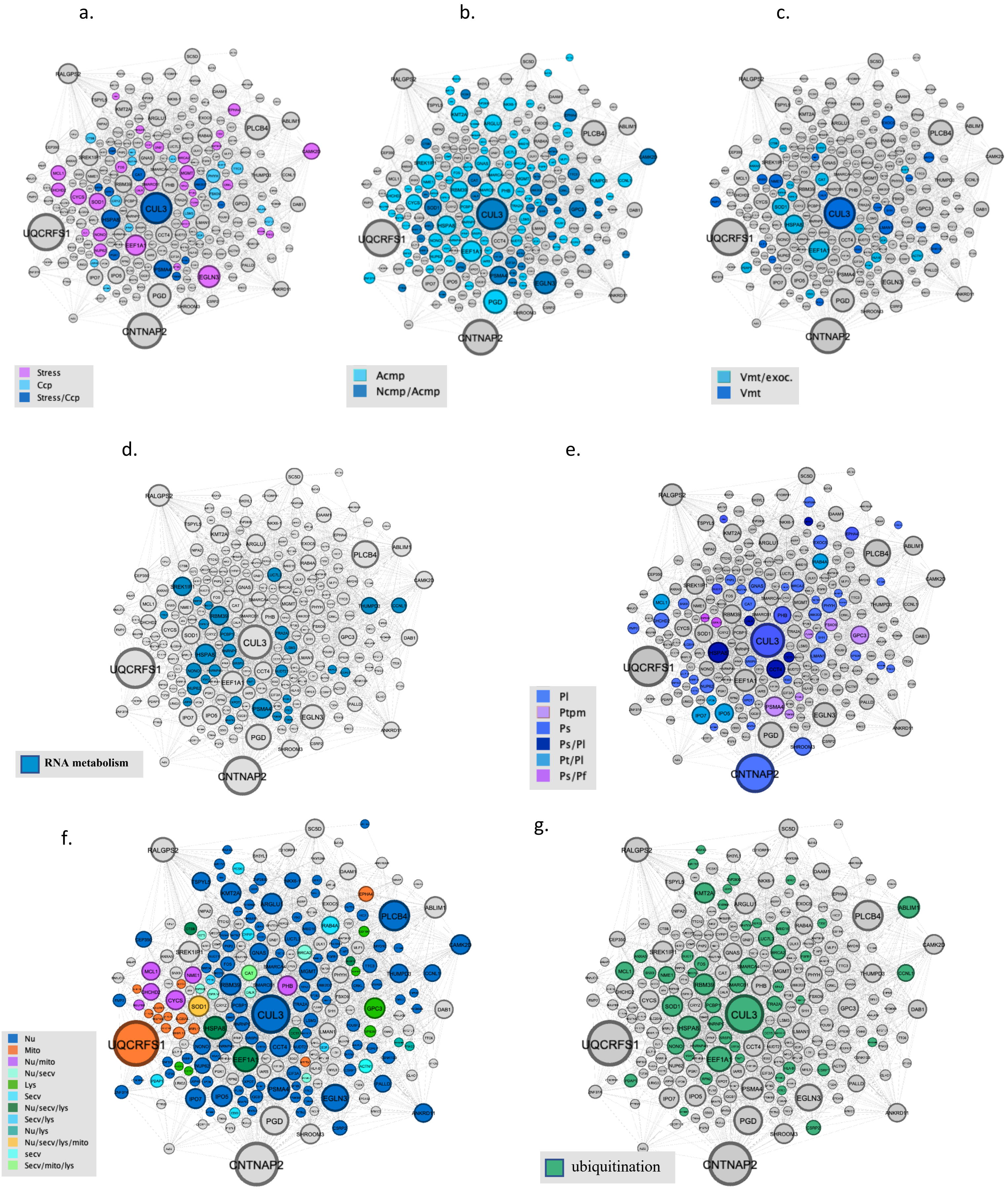

Supplementary Figure 7. Functional pathways significantly represented in the network. Functional pathways as obtained from String database. **a).** Reaction to stress, cell catabolic processes. **b).** nitrogen and aromatic compound metabolism. **c).** Vesicle mediated transport & exocytosis. **d).** RNA metabolism **e).** Protein metabolism. **f).** Localization. **g).** ubiquitination. (Supplementary Data 3). The network is based on 292 DEGs.

Abbreviations used in Supplementary Figure 7f.

**Acmp**	aromatic compound metabolic process (par of Ncmp)
**ccp**	cellular catabolic process
**exoc**	exocytosis
**lys**	lysosome
**mito**	mitochondrial
**Ncmp**	nitrogen compound metabolic process
**Nu**	nucleus
**Pf**	protein folding
**Pl**	protein localization
**Pn-m**	Metabolism of proteins
**Ps**	protein stability
**Pt**	protein transporter
**Ptpm**	post-translational protein modification
**RNAm**	Metabolism of RNA
**RNAp**	RNA processing
**secv**	secretory vesicle
**stress**	cellular response to stress
**Ubi**	Ubl conjugation
**Vmt**	vesicle-mediated transport

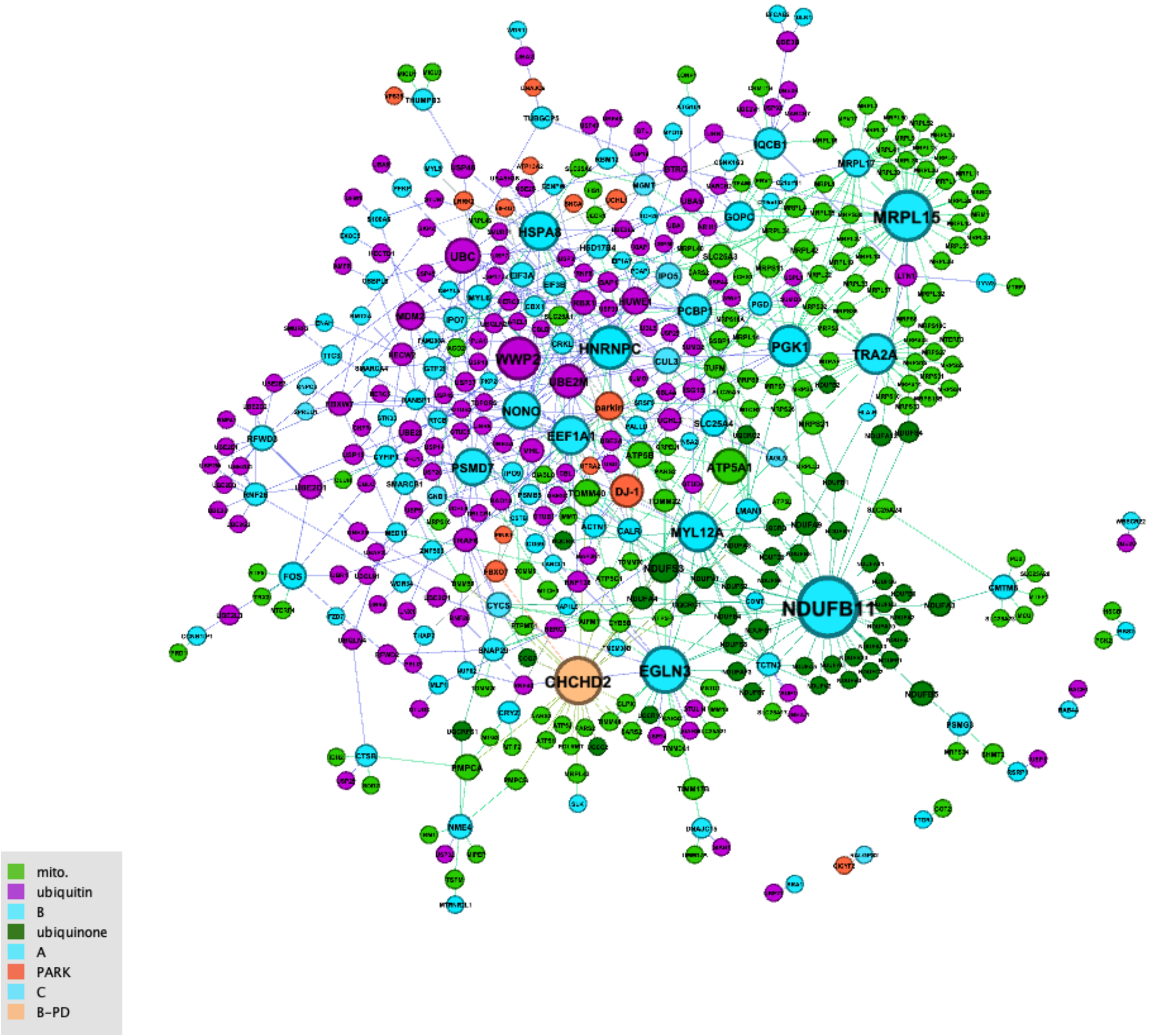

Supplementary Figure 8. The interaction of genes from Groups A-C with ubiquitination and mitochondrial genes. Only direct contacts of the differentially expressed genes (DEGs) in groups A-C were used. The graph illustrates which ubiquitination and mitochondrial genes the DEGs of the network interact with. DEGs are in light blue, ubiquitination genes they interact with are in purple, mitochondrial genes the DEGs interact with are in green and PARK genes are in orange. This figure illustrated that the DEGs interact with a vast array of ubiquitination and mitochondrial genes and that many of these interactions are common to other PARK genes, hence the effect of their differential expression, likely greatly impacts these networks, and that other PARK mutations have the capacity to impact the same network. The size of the nodes is based on the Betweenness Centrality calculation by the Gephi application, which roughly represents the connectedness of the node, hence the more nodes a particular node interacts with, the greater its size.

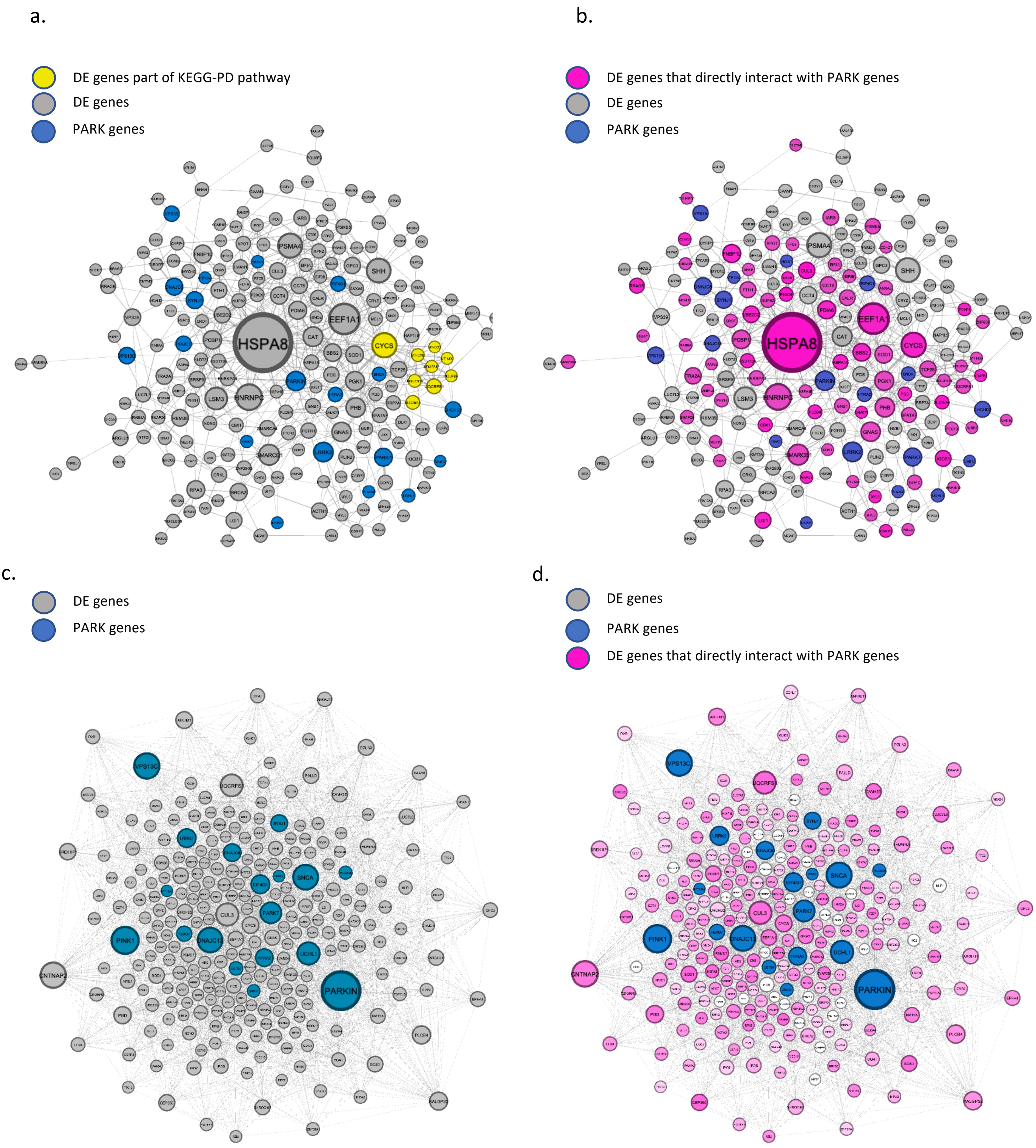

Supplementary Figure 9. Parkinson-associated genes (PARK genes) integrated into the network. Only direct interactions to genes known to be associated with PD (Supplementary Table 5) were included, so that only PD genes that directly interact with DE genes would be retained. The network is based on 292 DEGs. Figure a and b are based on STRING data, while c and d are based on GENEMANIA. The size of the nodes reflects the relative connectivity of that node to other DEGs, with larger nodes connected to more DEGs. **a).** PD genes as they integrate into the network through direct interactions with genes of the network. DE expressed genes that are part of the KEGG-PD pathway are highlighted in yellow **b).** The nearest DEG neighbour of each PARK gene was colored in pink. The darker the color, the more PARK genes the DE genes interacts with. HSPA8 is one of the most highly DE genes and also plays an important role in PD, it connects to several of the PARK genes. **c).** Interactions based on GeneMANIA. **d).** Genes that directly interact with PARK genes through interactions listed in GeneMANIA are colored in pink. The size of the

nodes is based on the Betweenness Centrality calculation by the Gephi application, which roughly represents the connectedness of the node, hence the more nodes a particular node interacts with, the greater its size.

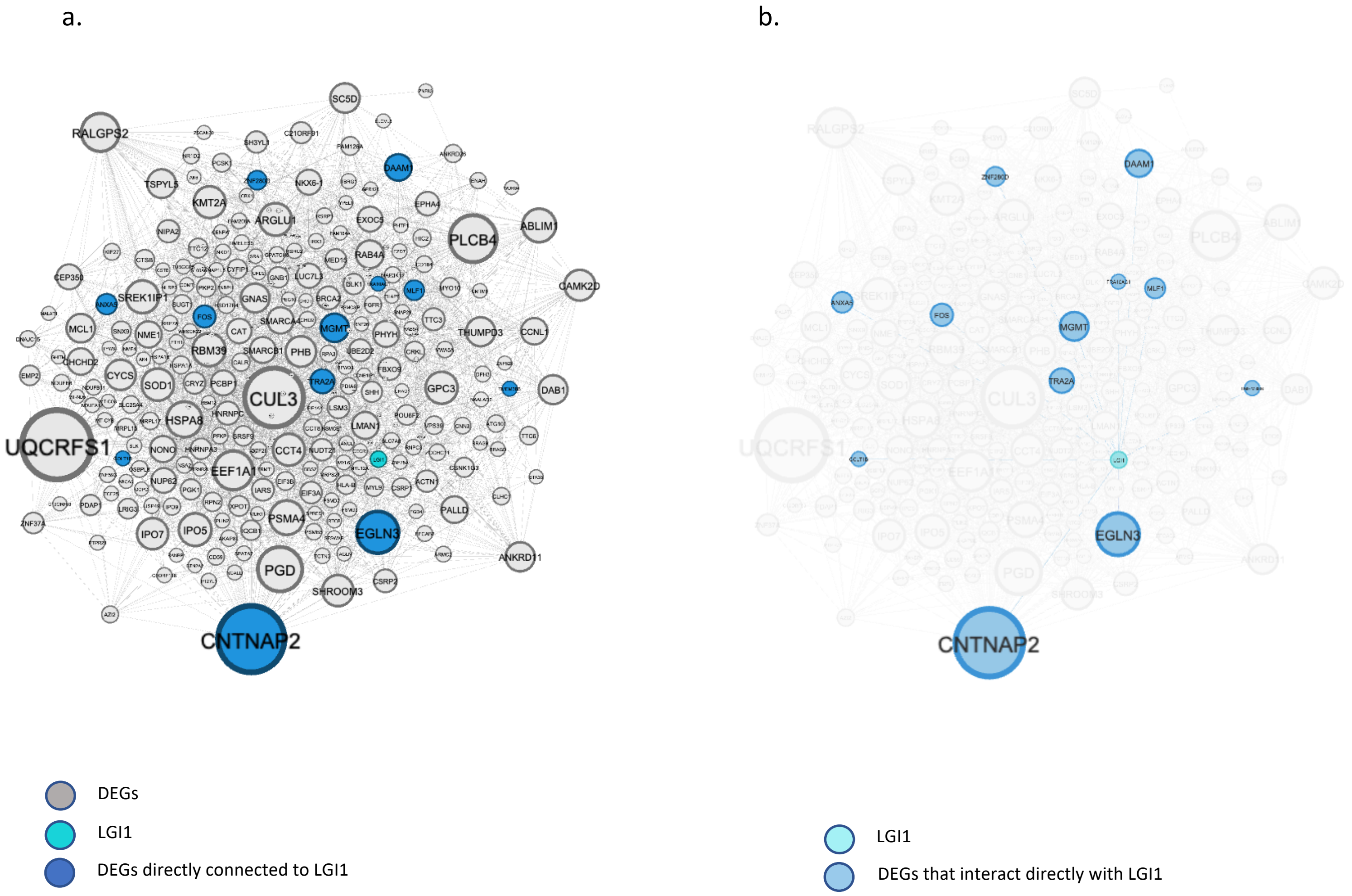

Supplementary Figure 10. Nearest neighbour of LGI1. The network is based on 292 DEGs.

a) LGI1[25] and its direct contacts, as seen within the DEG network.

b) LGI1 and its direct contacts selected.

The size of the nodes is based on the Betweenness Centrality calculation by the Gephi application, which roughly represents the connectedness of the node, hence the more nodes a particular node interacts with, the greater its size.

Alignment of sequenced fragment

primer used: PINK1_cDNA2_fwd   **Query** = NM_032409.2   **Sbjct** = PCR fragment

```
Query  924   TTCTCCGCGCCTTCACCTCTTCCGTGCCGCTGCTGCCAGGGGCCCTGGTCGACTACCCTG  983
             ||||||||||||||||||||||||||||||||||||||||||||||||||||||||||||
Sbjct  1     TTCTCCGCGCCTTCACCTCTTCCGTGCCGCTGCTGCCAGGGGCCCTGGTCGACTACCCTG  60

Query  984   ATGTGCTGCCCTCACGCCTCCACCCTGAAGGCCTGGGCCATGGCCGGACGCTGTTCCTCG  1043
             ||||||||||||||||||||||||||||||||||||||||||||||||||||||||||||
Sbjct  61    ATGTGCTGCCCTCACGCCTCCACCCTGAAGGCCTGGGCCATGGCCGGACGCTGTTCCTCG  120

Query  1044  TTATGAAGAACTATCCCTGTACCCTGCGCCAGTACCTTTGTGTGAACACACCCAGCCCCC  1103
             ||||||||||||||||||||||||||||||||||||||||||||||||||||||||||||
Sbjct  121   TTATGAAGAACTATCCCTGTACCCTGCGCCAGTACCTTTGTGTGAACACACCCAGCCCCC  180

Query  1104  GCCTCGCCGCCATGATGCTGCTGCAGCTGCTGGAAGGCGTGGACCATCTGGTTCAACAGG  1163
             ||||||||||||||||||||||||||||||||||||||||||||||||||||||||||||
Sbjct  181   GCCTCGCCGCCATGATGCTGCTGCAGCTGCTGGAAGGCGTGGACCATCTGGTTCAACAGG  240

Query  1164  GCATCGCGCACAGAGACCTGAAATCCGACAACATCCTTGTGGAGCTGGACCCAGACGGCT  1223
             ||||||||||||||||||||||||||||||||| ||||||||||||||||||||||||||
Sbjct  241   GCATCGCGCACAGAGACCTGAAATCCGACAACAACCTTGTGGAGCTGGACCCAGACGGCT  300

Query  1224  GCCCCTGGCTGGTGATCGCAGATTTTGGCTGCTGCCTGGCTGATGAGAGCATCGGCCTGC  1283
             ||||||||||||||||||||||||||||||||||||||||||||||||||||||||||||
Sbjct  301   GCCCCTGGCTGGTGATCGCAGATTTTGGCTGCTGCCTGGCTGATGAGAGCATCGGCCTGC  360

Query  1284  AGTTGCCCTTCAGCAGCTGGTACGTGGATCGGGGCGGAAACGGCTGTCTGATGGCCCCAG  1343
             ||||||||||||||||||||||||||||||||||||||||||||||||||||||||||||
Sbjct  361   AGTTGCCCTTCAGCAGCTGGTACGTGGATCGGGGCGGAAACGGCTGTCTGATGGCCCCAG  420

Query  1344  AGGTGTCCACGGCCCGTCCTGGCCCCAGGGCAGTGATTGACTACAGCAAGGCTGATGCCT  1403
             ||||||||||||||||||||||||||||||||||||||||||||||||||||||||||||
Sbjct  421   AGGTGTCCACGGCCCGTCCTGGCCCCAGGGCAGTGATTGACTACAGCAAGGCTGATGCCT  480

Query  1404  GGGCAGTGGGAGCCATCGCCTATGAAATCTTCGGGCTTGTCAATCCCTTCTA  1455
             |||||||||||||||||||||||||||||||||||||||||||||||| |||
Sbjct  481   GGGCAGTGGGAGCCATCGCCTATGAAATCTTCGGGCTTGTCAATCCCTCCTA  532
```

Supplementary Figure 11. Sequencing results showing homozygosity of the PINK1cell line for the ILE368ASN mutation. This sequence has been deposited to NCBI under the accession OK050183.1

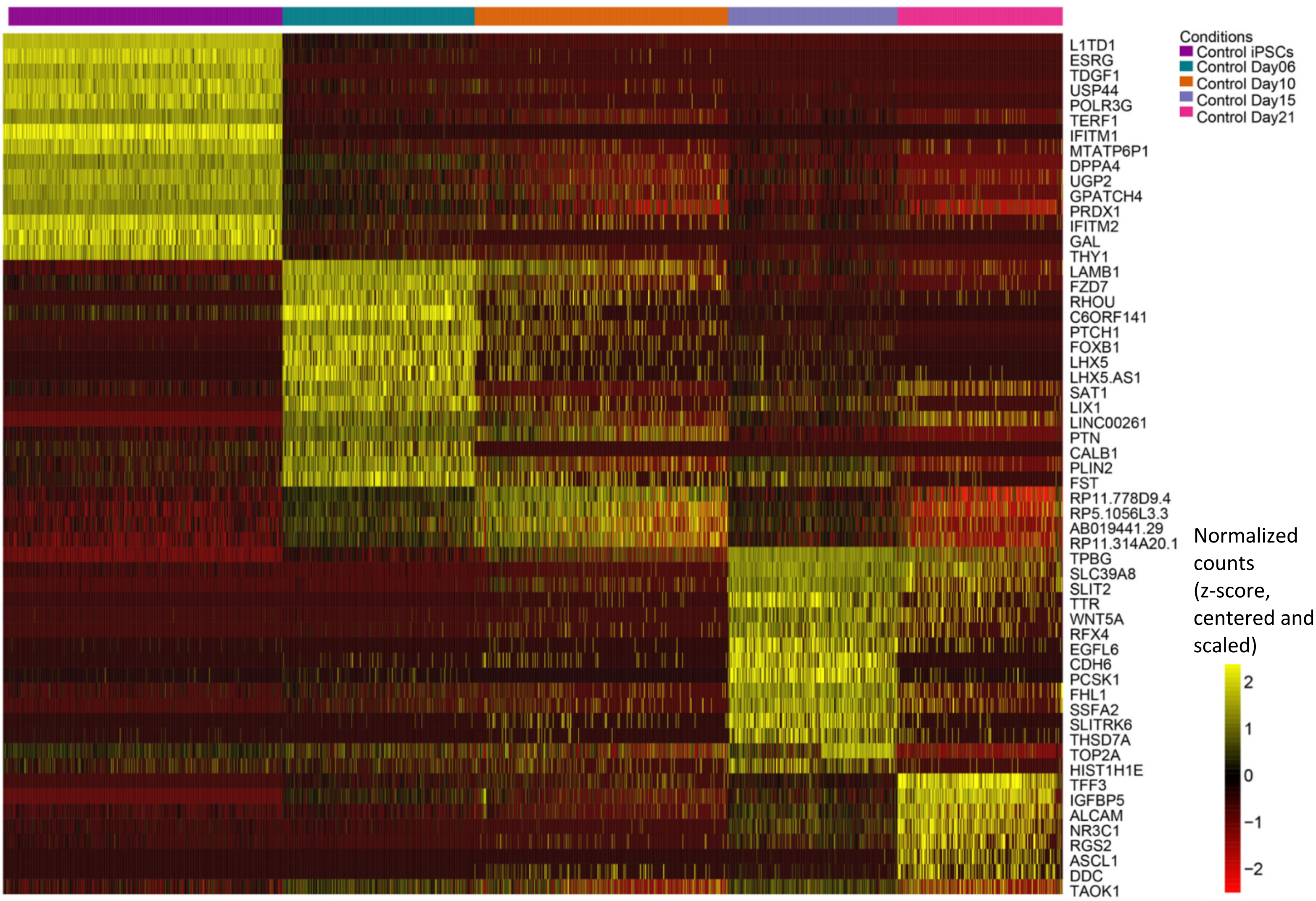

Supplementary Figure 12. Heatmap showing a gradual transition of gene expression between differentiation stages. Timepoints D0 (iPSCs), D6, D10, D15 and D21. As expected, D6 and D10 are quite similar, since there is only a small change in the differentiation factors the cells are exposed to (Supp. Table 1). The analysis is based on gene expression matrix of 4495 cells (39,194 genes). Colors correlate to normalized counts (z-score, centered and scaled) of indicated gene relative to the undifferentiated reference set, or for the iPSC group, relative to differentiated cells. As described in the text, expression of *TDGF-1*, *L1TD1*, *USP44*, *POLR3G*, *TERF1*, *IFITM1*, *DPPA4*, and *PRDX1* is associated with stemness.

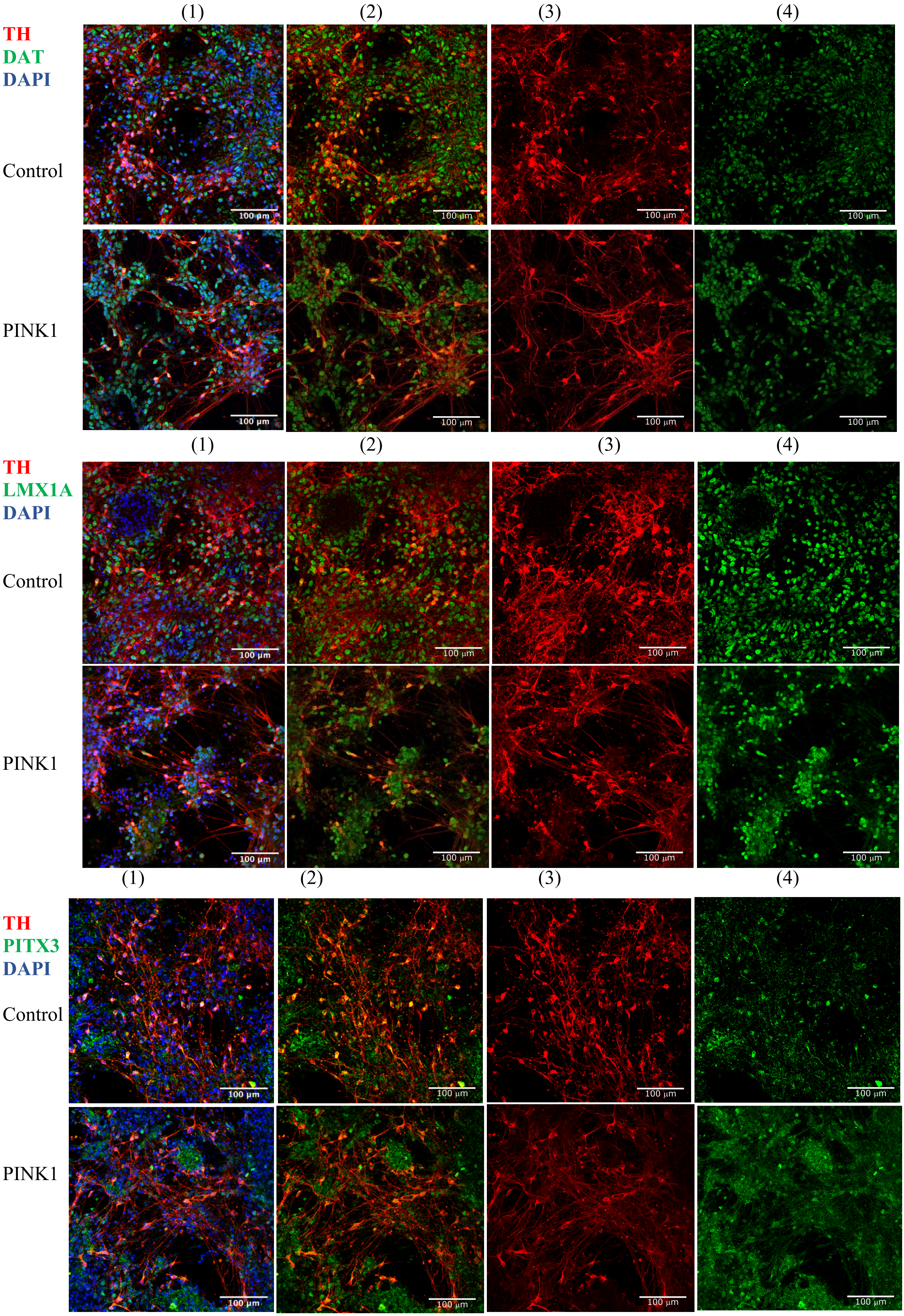

(1)
(2)
(3)
(4)
TH
DAT
DAPI
Control
PINK1
TH
LMX1A
DAPI
Control
PINK1
TH
PITX3
DAPI
Control
PINK1
100 µm

Supplementary Figure 13. Staining images of PINK1 and control cell lines at D35.

(1) TH (red); DAT/LMX1A/PITX3 (green); DAPI (blue)
(2) TH (red); DAT/LMX1A/PITX3 (green)
(3) TH (red)
(4) DAT/LMX1A/PITX3 (green)

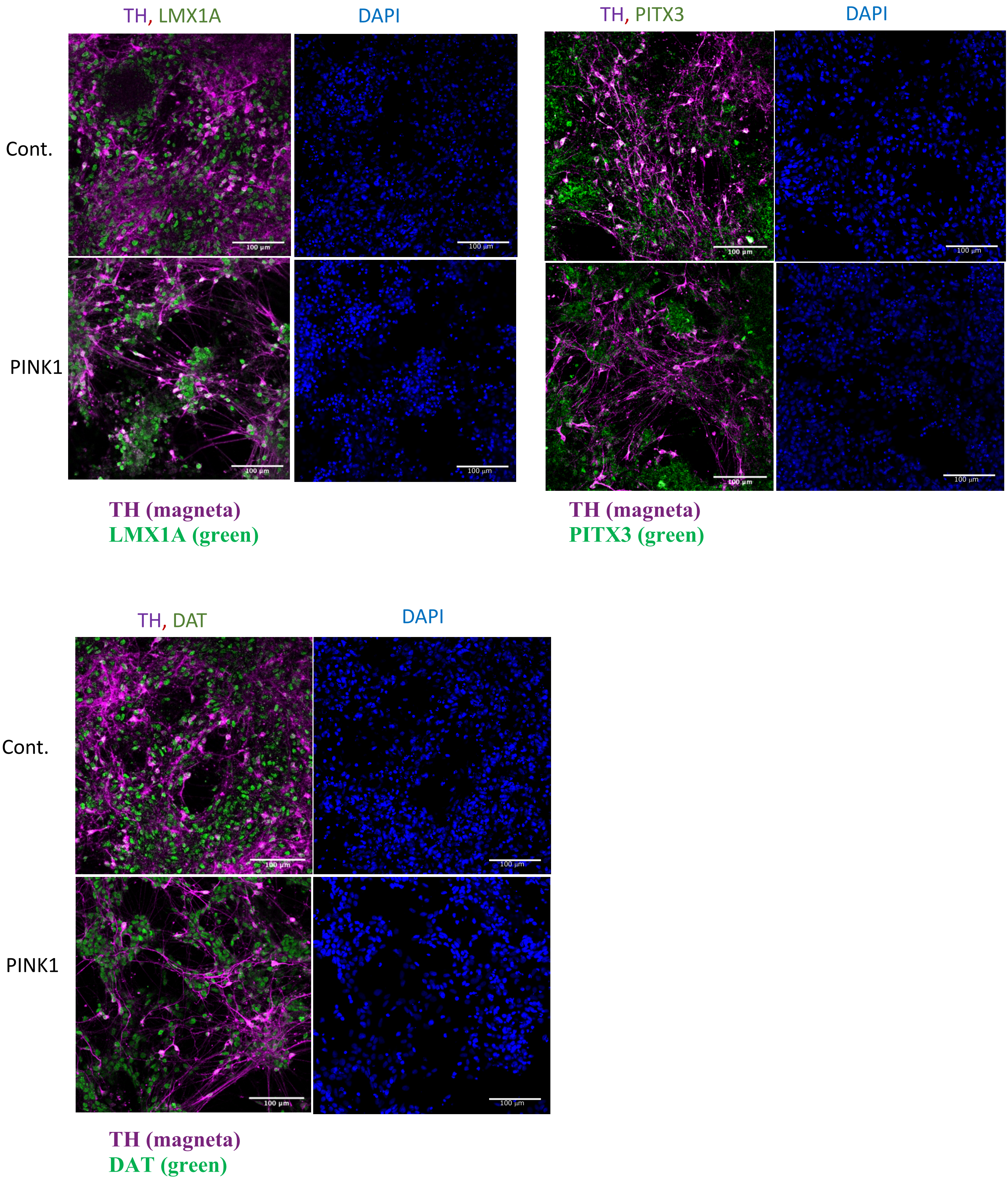

Supplementary Figure 14. images of PINK1 and control cell lines at D35 adapted using color blind-friendly palette.

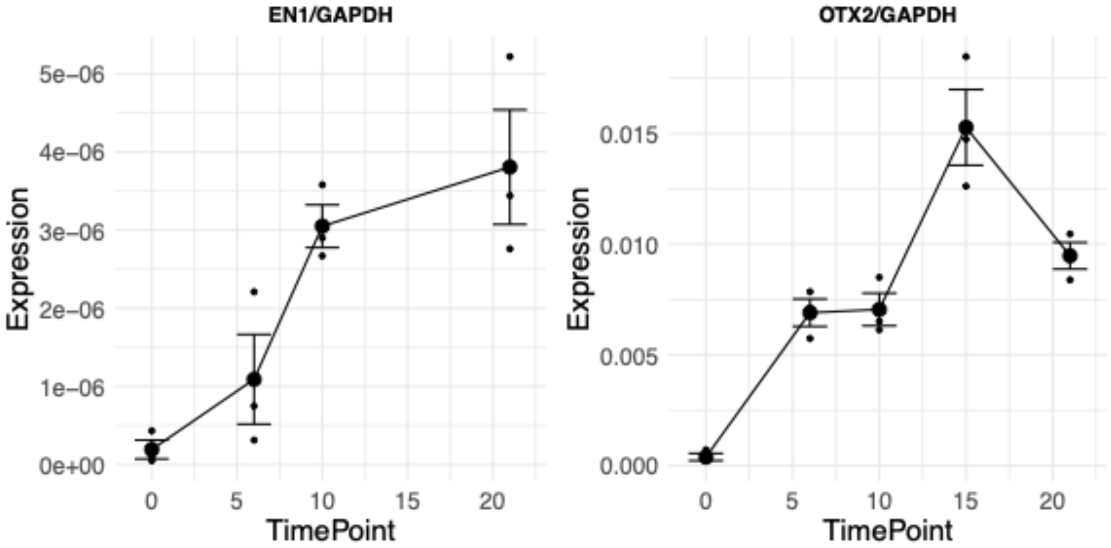

Supplementary Figure 15. Expression of the transcription factor EN1 and OTX2. Quantitation using absolute quantitation via qPCR and standardization to a housekeeping gene. Each timepoint represents three biological replicates, amplified in duplicate. Standard error (SE) bars are the SE of biological replicates. The expression levels are standardized to total RNA and to the expression of the housekeeping gene GAPDH. This illustrates why EN1 is not detected by SC-RNAseq, due to the loss of information regarding genes expressed at a low level as a result of data pre-processing. Input data is listed in Supplementary Data 7.

a.

b.

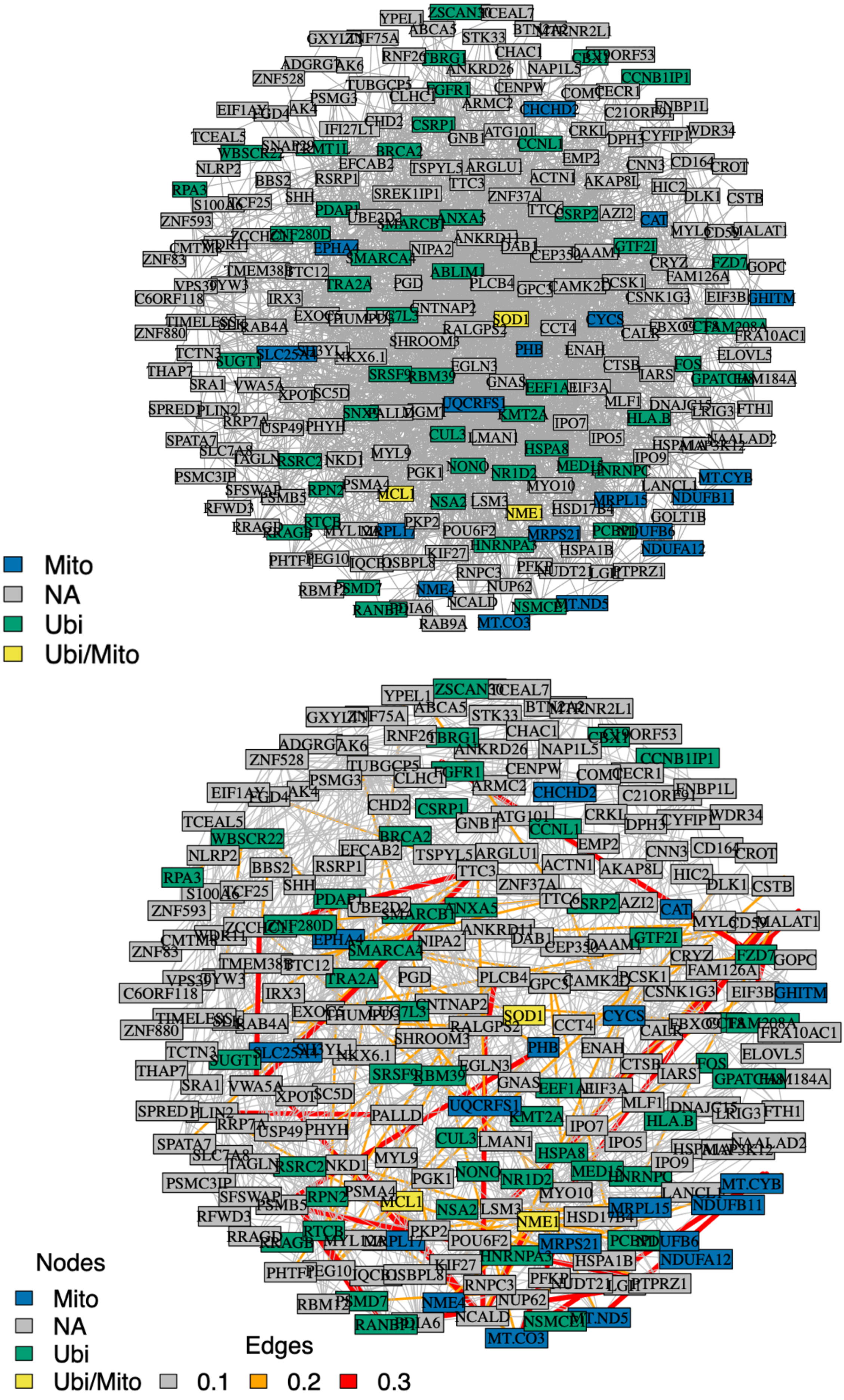

Supplementary Figure 16. Network analysis. a). Protein-protein interaction information obtained from the STRING and GeneMANIA databases. **b).** A correlation network based on the correlation of expression of DEGs (p-value < 0.05, correlation > 0.1). The analysis is based on gene expression matrix of 4495 cells (39,194 genes). The network is based on 292 DEGs.

a.

b.

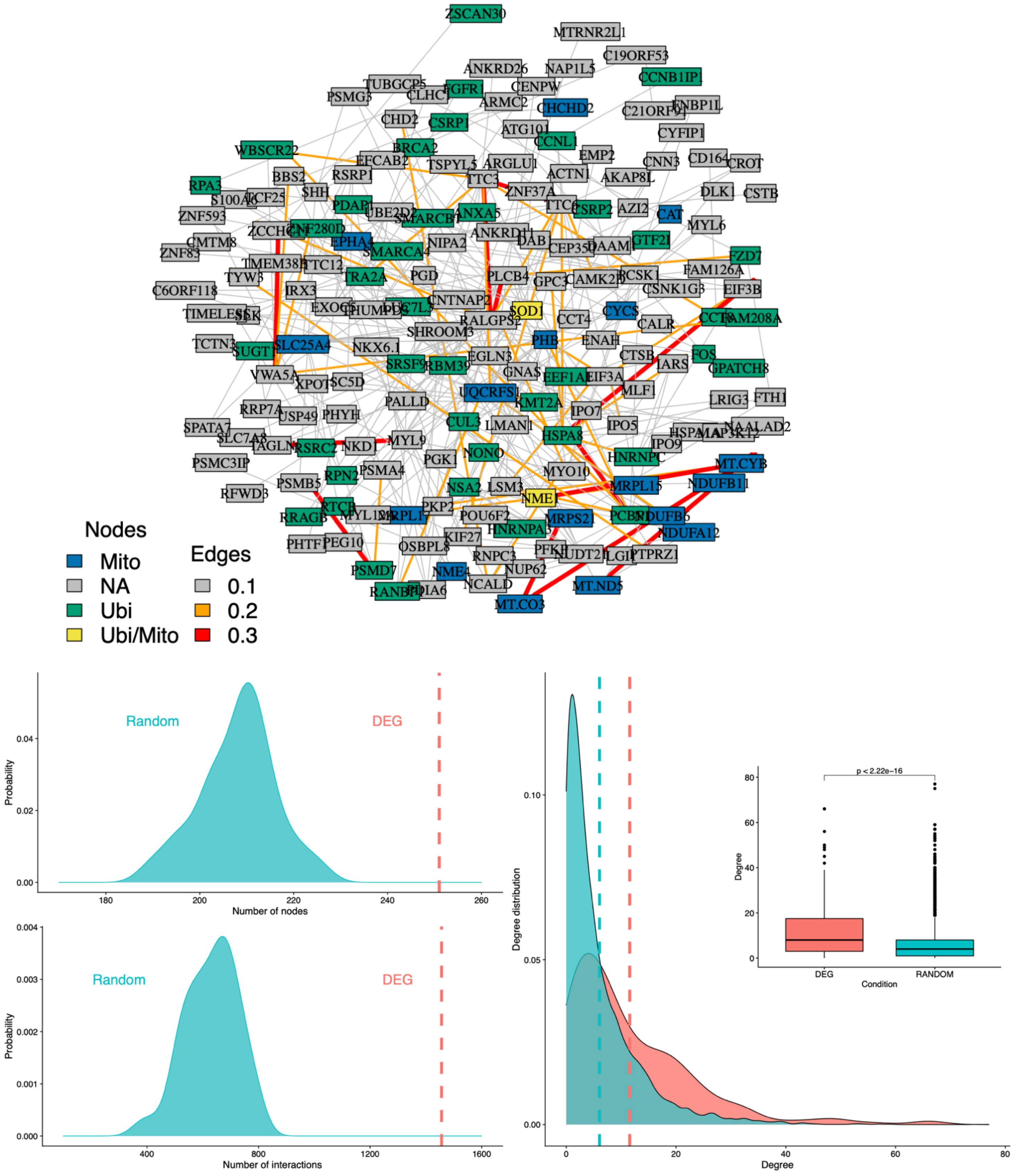

Supplementary Figure 17. Network analysis. **a).** We identified edges common between network a) and b). This network consists of 860 interactions. We then extracted shared interactions of these two networks, which yielded 297 interactions. **b).** In order to validate the PPI network produced by STIRNGdb (v10), we created 50 PPI (protein-protein interaction) networks using 292 random genes (same as the number of DEGs). We then compared the number of detected proteins, the number of interactions between the genes and the distribution of the node degrees. Box Plot depicts the number of degrees through their quartiles per group (Red: ppi network based on the differential expressed genes, Blue: ppi networks of random genes). Wilcoxon test performed to access if the two-degree distributions are different from one another in a statistically significant manner, which showed a statistically significant difference (p=2.22e-16).

a.

SERVICE REPORT

YALE STEM CELL CENTER hESC/iPSC CORE FACILITY

Service requested: iPSC generation.

REPORT DETAIL

Dated: 11/20/2015

Mycoplasma Test: Negative

Description of Starting Somatic Cell Growth: Normal

Reprogramming description:

- Reprogramming factor delivery Method: Sendai virus

-IPSC Culture Conditions: iPSC clones were cultured in mTeSR medium on GFR-Matrigel coated plates, and passed by dispase.

Cell Lines	iPSC clones	# of vials	# of Shippment	Note
ND40066	Clone-1	3	3	
	Clone-2	3	3	
	**Clone-8**	**3**	**3**	
	Clone-10	3	3	
	Clone-18	3	3	
	Clone 3	1	1	**Back up**
	Clone 4	1	1	
	Clone 5	1	1	

b.

cell line	Reading A	Reading B	Ratio		
ND29542-7	223	178	0.80	negative	
ND29542-3	253	135	0.53	negative	
ND40996-10	185	66	0.36	negative	
ND40996-7	348	115	0.33	negative	
ND40996-1	160	58	0.36	negative	
**ND40066-8**	**203**	**72**	**0.35**	**negative**	
ND40066-7	203	59	0.29	negative	
ND40066-2	285	65	0.23	negative	
ND34267-8	205	125	0.61	negative	
-C (buffer)	623	57	0.09	negative	
+C (kit)	436	33057	75.82	positive	
				less than 1 = negative	
MycoAlertTM Assay Control Set LT07-518					
MycoAlertTM Mycoplasma Detection Kit LT07-418					

Supplementary Figure 18. Mycoplasma testing report. a. Mycoplasma testing report generated after the initial reprogramming of the iPSC clones by the Yale Stem Cell Center Core Facility. **b.** The ND40088 clone 8 passage 15 (expanded from cells used in our experiments) was most recently tested for mycoplasma on 20.4.2021. -C is negative control (buffer only), +C is a positive control provided of the MycoAlert Control Set.

SUPPLEMENTARY REFERENCES

1. Tomishima, M. Midbrain dopamine neurons from hESCs. *StemBook* (2014) doi:10.3824/stembook.1.70.1.
2. Kriks, S. *et al.* Dopamine neurons derived from human ES cells efficiently engraft in animal models of Parkinson's disease. *Nature* (2011) doi:10.1038/nature10648.
3. Kriks, S. *et al.* Floor plate-derived dopamine neurons from hESCs efficiently engraft in animal models of PD. *Nature* (2012) doi:10.1038/nature10648.Floor.
4. Blaess, S. & Ang, S. L. Genetic control of midbrain dopaminergic neuron development. *Wiley Interdisciplinary Reviews: Developmental Biology* (2015) doi:10.1002/wdev.169.
5. Ang, S. L. Transcriptional control of midbrain dopaminergic neuron development. *Development* (2006) doi:10.1242/dev.02501.
6. Björklund, A. & Dunnett, S. B. Dopamine neuron systems in the brain: an update. *Trends in Neurosciences* (2007) doi:10.1016/j.tins.2007.03.006.
7. Tiklová, K. *et al.* Single-cell RNA sequencing reveals midbrain dopamine neuron diversity emerging during mouse brain development. *Nat. Commun.* (2019) doi:10.1038/s41467-019-08453-1.
8. Hegarty, S. V., Sullivan, A. M. & O'Keeffe, G. W. Midbrain dopaminergic neurons: A review of the molecular circuitry that regulates their development. *Developmental Biology* (2013) doi:10.1016/j.ydbio.2013.04.014.
9. Arenas, E., Denham, M. & Villaescusa, J. C. How to make a midbrain dopaminergic neuron. *Dev.* (2015) doi:10.1242/dev.097394.
10. Ásgrímsdóttir, E. S. & Arenas, E. Midbrain Dopaminergic Neuron Development at the Single Cell Level: In vivo and in Stem Cells. *Frontiers in Cell and Developmental Biology* (2020) doi:10.3389/fcell.2020.00463.
11. Klein, C. & Westenberger, A. Genetics of Parkinson's disease. *Cold Spring Harb. Perspect. Med.* (2012) doi:10.1101/cshperspect.a008888.
12. Puschmann, A. New Genes Causing Hereditary Parkinson's Disease or Parkinsonism. *Current Neurology and Neuroscience Reports* (2017) doi:10.1007/s11910-017-0780-8.
13. Kirola, L., Behari, M., Shishir, C. & Thelma, B. K. Identification of a novel homozygous mutation Arg459Pro in SYNJ1 gene of an Indian family with autosomal recessive juvenile Parkinsonism. *Park. Relat. Disord.* (2016) doi:10.1016/j.parkreldis.2016.07.014.
14. Quadri, M. *et al.* Mutation in the SYNJ1 gene associated with autosomal recessive, early-onset parkinsonism. *Hum. Mutat.* (2013) doi:10.1002/humu.22373.
15. Olgiati, S. *et al.* PARK20 caused by SYNJ1 homozygous Arg258Gln mutation in a new Italian family. *Neurogenetics* (2014) doi:10.1007/s10048-014-0406-0.
16. Funayama, M. *et al.* CHCHD2 mutations in autosomal dominant late-onset Parkinson's disease: A genome-wide linkage and sequencing study. *Lancet Neurol.* (2015) doi:10.1016/S1474-4422(14)70266-2.
17. Lesage, S. *et al.* Loss of VPS13C Function in Autosomal-Recessive Parkinsonism Causes Mitochondrial Dysfunction and Increases PINK1/Parkin-Dependent Mitophagy. *Am. J. Hum. Genet.* (2016) doi:10.1016/j.ajhg.2016.01.014.
18. Tomishima, M. *StemBook*. (Harvard Stem Cell Institute, 2012). doi:10.3824/stembook.1.70.1.
19. Szklarczyk, D. *et al.* STRING v11: Protein-protein association networks with increased coverage, supporting functional discovery in genome-wide experimental datasets. *Nucleic Acids Res.* (2019) doi:10.1093/nar/gky1131.
20. Qiu, B. *et al.* DJ-1 promotes development of DEN-induced hepatocellular carcinoma and proliferation of liver cancer cells. *Oncotarget* (2017) doi:10.18632/oncotarget.14293.
21. Rouillard, A. D. *et al.* The harmonizome: a collection of processed datasets gathered to serve and mine knowledge about genes and proteins. *Database (Oxford).* (2016) doi:10.1093/database/baw100.
22. Oliveira, L. M. A. *et al.* Elevated α-synuclein caused by SNCA gene triplication impairs neuronal differentiation and maturation in Parkinson's patient-derived induced pluripotent stem cells. *Cell Death Dis.* (2015) doi:10.1038/cddis.2015.318.
23. Cruz-Monteagudo, M. *et al.* Efficient and biologically relevant consensus strategy for Parkinson's disease gene prioritization. *BMC Med. Genomics* (2016) doi:10.1186/s12920-016-0173-x.
24. Volakakis, N. *et al.* Nurr1 and retinoid X receptor ligands stimulate ret signaling in dopamine neurons

and can alleviate α-synuclein disrupted gene expression. *J. Neurosci.* (2015) doi:10.1523/JNEUROSCI.1155-15.2015.

25. Kurtis, M. M., Toledano, R., García-Morales, I. & Gil-Nagel, A. Immunomodulated parkinsonism as a presenting symptom of LGI1 antibody encephalitis. *Parkinsonism and Related Disorders* (2015) doi:10.1016/j.parkreldis.2015.08.014.
