# Supplementary material for: Single-cell transcriptomics of human iPSC differentiation dynamics reveal a core molecular network of Parkinson’s disease
Source: Commun Biol. 2022 Jan 13;5:49. doi: 10.1038/s42003-021-02973-7 (PMC8758783; doi:10.1038/s42003-021-02973-7)
Supplement: Supplementary file 3 — Description of Additional Supplementary Files [file 42003_2021_2973_MOESM3_ESM.pdf]

Description of Additional Supplementary Files

File name: Supplementary Data 1.
Description: Group D, 284 genes. Repeating the analysis as in Group C, but using only timepoints D6, D15 and D21 identified a total of 286 DEGs (Group D). In black: genes of group D only. In blue: genes of group D that are also part of group C. In green: six genes of group C that were not included in Group D, hence, even though group D consists of 286 genes, the total number used in the network analysis was 292.

File name: Supplementary Data 2.
Description: Correlation interactions.

File name: Supplementary Data 3.
Description: Pathway association. Pathway association was Page 8 of 23 obtained from STRING18, except for PD. PD association was obtained through a manual search and references are listed in Supp. Table 10.

File name: Supplementary Data 4.
Description: PD association. Manual literature search was used to identify association with PD of the 291 genes identified in this study. "Excluded" category lists the reason these genes do not appear in the protein-protein interaction network. "G" = group the genes belong to.

File name: Supplementary Data 5.
Description: KEGG pathways associated with this dataset, output generated by STRING18 database.

File name: Supplementary Data 6.
Description: Mycoplasma testing results.

File name: Supplementary Data 7.
Description: Data input for qPCR figures. This data was used to generate Fig. 3 and Supplementary Fig. 15.

File name: Supplementary Data 8.
Description: Fig 3e metadata.

File name: Supplementary Data 9.
Description: Data used to generate Fig. 3e table.

File name: Supplementary Data 10.
Description: Data used to generate Fig. 7a.
