# Supplementary material for: Single-cell transcriptomics of human iPSC differentiation dynamics reveal a core molecular network of Parkinson’s disease
Source: Commun Biol. 2022 Jan 13;5:49. doi: 10.1038/s42003-021-02973-7 (PMC8758783; doi:10.1038/s42003-021-02973-7)
Supplement: Supplementary file 14 — Reporting Summary [file 42003_2021_2973_MOESM14_ESM.pdf]

nature research

Corresponding author(s): Gabriela Novak
Alexander Skupin

Last updated by author(s): Sep 9, 2021

# Reporting Summary

Nature Research wishes to improve the reproducibility of the work that we publish. This form provides structure for consistency and transparency in reporting. For further information on Nature Research policies, see our Editorial Policies and the Editorial Policy Checklist.

## Statistics

For all statistical analyses, confirm that the following items are present in the figure legend, table legend, main text, or Methods section.

| n/a | Confirmed | |
|---|---|---|
| ☐ | ☒ | The exact sample size ($n$) for each experimental group/condition, given as a discrete number and unit of measurement |
| ☐ | ☒ | A statement on whether measurements were taken from distinct samples or whether the same sample was measured repeatedly |
| ☐ | ☒ | The statistical test(s) used AND whether they are one- or two-sided<br>*Only common tests should be described solely by name; describe more complex techniques in the Methods section.* |
| ☐ | ☒ | A description of all covariates tested |
| ☐ | ☒ | A description of any assumptions or corrections, such as tests of normality and adjustment for multiple comparisons |
| ☐ | ☒ | A full description of the statistical parameters including central tendency (e.g. means) or other basic estimates (e.g. regression coefficient) AND variation (e.g. standard deviation) or associated estimates of uncertainty (e.g. confidence intervals) |
| ☐ | ☒ | For null hypothesis testing, the test statistic (e.g. $F$, $t$, $r$) with confidence intervals, effect sizes, degrees of freedom and $P$ value noted<br>*Give P values as exact values whenever suitable.* |
| ☒ | ☐ | For Bayesian analysis, information on the choice of priors and Markov chain Monte Carlo settings |
| ☒ | ☐ | For hierarchical and complex designs, identification of the appropriate level for tests and full reporting of outcomes |
| ☒ | ☐ | Estimates of effect sizes (e.g. Cohen's $d$, Pearson's $r$), indicating how they were calculated |

*Our web collection on statistics for biologists contains articles on many of the points above.*

## Software and code

Policy information about availability of computer code

| | |
|---|---|
| Data collection | All data is original, collected from our samples. All analysis scripts will be publicly available by the open and sustained gitlab repository https://gitlab.lcsb.uni.lu/ICS-lcsb/ipscs_pink1 |
| Data analysis | The cDNA libraries were prepared using the standard Nextera XT tagmentation kit (Illumina).<br>Purified Drop-seq cDNA libraries were sequenced using Illumina NextSeq 500 with the recommended sequencing protocol except for 6pM of custom primer (GCCTGTCCGCGGAAGCAGTGGTATCAACG CAGAGTAC) applied for priming of read 1.<br>The FASTQ files were assembled from the raw BCL files using Illumina's bcl2fastq converter and run through the FASTQC codes (Babraham bioinformatics; https://www.bioinformatics.babraham.ac.uk/projects/fastqc/)<br>The FASTQ files were then merged and converted into binaries using PICARD's FastqToSam algorithm.<br>The sequencing reads were converted into a digital gene expression matrix using the Drop-seq bioinformatics pipeline (Ref.37).<br>The integration of the filtered matrices of the different datasets was performed using scTransform (Ref.127) on a Seurat object (Ref.128). |

For manuscripts utilizing custom algorithms or software that are central to the research but not yet described in published literature, software must be made available to editors and reviewers. We strongly encourage code deposition in a community repository (e.g. GitHub). See the Nature Research guidelines for submitting code & software for further information.

## Data

Policy information about availability of data

All manuscripts must include a data availability statement. This statement should provide the following information, where applicable:

- Accession codes, unique identifiers, or web links for publicly available datasets
- A list of figures that have associated raw data
- A description of any restrictions on data availability

Single cell RNAseq data is available through the Gene Expression Omnibus (GEO), accession  number GSE183248. https://www.ncbi.nlm.nih.gov/geo/query/acc.cgi?acc=GSE183248
The proteomics data is available via the Proteomics Identification Database (PRIDE). https://r3lab.uni.lu/frozen/cca2-s098
Code availability. All analysis scripts are be publicly available via: https://gitlab.lcsb.uni.lu/ICS-lcsb/ipscs_pink1
All other data is available in Supplementary Dara Sets.

# Field-specific reporting

Please select the one below that is the best fit for your research. If you are not sure, read the appropriate sections before making your selection.

☒ Life sciences ☐ Behavioural & social sciences ☐ Ecological, evolutionary & environmental sciences

For a reference copy of the document with all sections, see nature.com/documents/nr-reporting-summary-flat.pdf

# Life sciences study design

All studies must disclose on these points even when the disclosure is negative.

| | |
|---|---|
| Sample size | The final gene expression matrix, which was used for the downstream analysis, consisted of 4495 cells and 39,194 genes with a median total number of mRNA counts of 7750 and a median number of expressed genes of 3521. |
| Data exclusions | The following is listed in the Methods section, where appropriate references were included:<br>The identification of low quality cells was done separately for each data set. In order to select only the highest quality data, we sorted the cells by their cumulative gene expression. Only cells with the highest cumulative expression were considered for the analysis.<br>In addition to this filtering we defined cells as low-quality based on three criteria for each cell. The number of expressed genes must be more than 200 and 2 median-absolute- deviations (MADs) above the median; the total number of counts has to be 2 MADs above or below the median, and the percentage of counts to mitochondrial genes has to be 1.5 MADs above the median. Cells failing at least one criteria were considered as low quality cells and filtered out from further analysis. Similar to the cell filtering, we filtered out low quality genes, identified by being expressed in less than 10 cells in the data. The integration of the filtered matrices of the different datasets was performed using scTransform on a Seurat object128 based on the treatment. The final gene expression matrix, which was used for the downstream analysis, consisted of 4495 cells and 39,194 genes with a median total number of mRNA counts of 7750 and a median number of expressed genes of 3521. Principal component analysis (PCA) was computed using the 5000 most variable genes of the integrated data. The clustering of data was performed using Louvain clustering. The resolution of the clustering was selected based on the best silhouette score of the different resolutions.  A short list of manually curated markers was used to validate the different stages of the differentiation process. |
| Replication | We performed differential expression analysis between two treatments (control and PINK1), in a pairwise manner, comparing PINK1 and a control cell line at each of four time-points. Each time-point was generated independently, differentiated in a separate differentiation round, and hence with cells of different passage number. Only genes, differential expression of which was replicated in all independently generated pairs, were retained. The differential expression analysis was done using MAST (default parameters) on the normalized counts using the total number of transcripts in each cell as a covariate and the Bonferroni correction to correct for multiple hypothesis testing (Padj). In addition, we tried to find conserved markers among the different time points using MAST again and the total number of transcripts in each cell as a latent variable. Genes with fold changes of the same sign in the fold change were then identified across the different time points and the average fold change was calculated. The genes with average fold change > 0.1 and maximum adjusted p-value < 0.01 were selected as differentially expressed. |
| Randomization | All samples were processed as a whole, there was no selection for a cell subtype. Selection of cells was random, by chance of fusion of a cell with a reagent droplet during the Dropseq experiment. |
| Blinding | Samples were processed at the same time, without attention to their source. |

# Reporting for specific materials, systems and methods

We require information from authors about some types of materials, experimental systems and methods used in many studies. Here, indicate whether each material, system or method listed is relevant to your study. If you are not sure if a list item applies to your research, read the appropriate section before selecting a response.

## Materials & experimental systems

| n/a | Involved in the study |
| --- | --- |
| ☐ | ☒ Antibodies |
| ☐ | ☒ Eukaryotic cell lines |
| ☒ | ☐ Palaeontology and archaeology |
| ☒ | ☐ Animals and other organisms |
| ☒ | ☐ Human research participants |
| ☒ | ☐ Clinical data |
| ☒ | ☐ Dual use research of concern |

## Methods

| n/a | Involved in the study |
| --- | --- |
| ☒ | ☐ ChIP-seq |
| ☒ | ☐ Flow cytometry |
| ☒ | ☐ MRI-based neuroimaging |

# Antibodies

Antibodies used

POU5F1 (Oct3/4) Santa Cruz Biotechnology sc-5279 - RRID AB_628051
Tra-1-60 Merck Millipore MAB4360 - RRID AB_2119183
PITX3 sigmaaldrich HPA044639 - RRID:AB_10964194
LMX1A abcam ab139726 - RRID:AB_2827684
Slc6A3/DAT Thermofisher PA1-4656 - RRID:AB_558680
MAP2 Sigma / Merck MAB3418 - RRID:AB_94856
TH Pel-Freez Biologicals P40101 - RRID:AB_2313713
TH Merck Millipore TH MAB 318 - RRID:AB_2201528
Donkey anti-Rabbit IgG, Alexa Fluor 488 Thermofisher A32790 - RRID:AB_2762833
Donkey anti-Mouse IgG, Alexa Fluor Plus 555 Thermofisher A32773 - RRID AB_2762848

Validation

All antibodies are standard commercial products validated by the manufacturer.
POU5F1 (Oct3/4) RRID AB_628051: 151 references available https://antibodyregistry.org/search.php?q=AB_628051
Tra-1-60 - RRID AB_2119183: 53 references available https://antibodyregistry.org/search.php?q=AB_2119183
PITX3 sigmaaldrich HPA044639 - RRID:AB_10964194: tested by manufacturer by (1) IHC tissue array of 44 normal human tissues and 20 of the most common cancer type tissues and (2) by protein array of 364 human recombinant protein fragments. https://www.sigmaaldrich.com/LU/en/product/sigma/hpa044639
LMX1A - RRID:AB_2827684: Candelario KM, Balaj L, Zheng T, Skog J, Scheffler B, Breakefield X, Schüle B, Steindler DA. Exosome/microvesicle content is altered in leucine-rich repeat kinase 2 mutant induced pluripotent stem cell-derived neural cells. J Comp Neurol. 2020 May;528(7):1203-1215. doi: 10.1002/cne.24819. Epub 2019 Nov 30. PMID: 31743443.
Slc6A3/DAT-RRID:AB_558680: Julku UH, Panhelainen AE, Tiilikainen SE, Svarcbahs R, Tammimäki AE, Piepponen TP, Savolainen MH, Myöhänen TT. Prolyl Oligopeptidase Regulates Dopamine Transporter Phosphorylation in the Nigrostriatal Pathway of Mouse. Mol Neurobiol. 2018 Jan;55(1):470-482. doi: 10.1007/s12035-016-0339-8. Epub 2016 Dec 13. PMID: 27966077.
MAP2 - RRID:AB_94856: 35 references available https://antibodyregistry.org/search.php?q=AB_94856
TH P40101 - RRID:AB_2313713- References: PMID:16871540, PMID:17245706, PMID:17990269, PMID:18537141, PMID:18973275, PMID:19655400, PMID:20653035, PMID:26850701, PMID:27716510, PMID:28840468, PMID:29315562, PMID:30104344, PMID:30840325, PMID:31099332
TH MAB 318 - RRID:AB_2201528 - large number of references, listed at https://antibodyregistry.org/search.php?q=AB_2201528
Donkey anti-Rabbit IgG, Alexa Fluor 488 Thermofisher A32790 http://antibodyregistry.org/AB_2762833; References: PMID:31386177, PMID:31665628, PMID:31951014, PMID:32488015
Donkey anti-Mouse IgG, Alexa Fluor Plus 555 Thermofisher A32773 - RRID AB_2762848. Gillingham AK, Bertram J, Begum F, Munro S. In vivo identification of GTPase interactors by mitochondrial relocalization and proximity biotinylation. Elife. 2019 Jul 11;8:e45916. doi: 10.7554/eLife.45916. PMID: 31294692; PMCID: PMC6639074.

# Eukaryotic cell lines

Policy information about cell lines

Cell line source(s)

Fibroblasts obtained from Coriell Institute repository, catalogue number ND40066

Authentication

Authentication was performed via STR analysis and is part of a manuscript we are submitting this month to Stem Cell Research - Lab Resource: Stem Cell Line:
ND40066-PINK1-ILE368ASN-clone 2 (LCSBi002-A)
ND40066-PINK1-ILE368ASN-clone 7 (LCSBi002-B)
ND40066-PINK1-ILE368ASN-clone 8 (LCSBi002-C)

Mycoplasma contamination

All cell lines were tested for mycoplasma and were shown to be negative.

Commonly misidentified lines
(See ICLAC register)

none
